# Mutation of the RelA(p65) Thr505 phosphosite disrupts the DNA replication stress response leading to CHK1 inhibitor resistance

**DOI:** 10.1042/BCJ20220089

**Published:** 2022-10-14

**Authors:** Jill E. Hunter, Amy E. Campbell, Jacqueline A. Butterworth, Helene Sellier, Nicola L. Hannaway, Saimir Luli, Achilleas Floudas, Niall S. Kenneth, Adam J. Moore, Philip J. Brownridge, Huw D. Thomas, Jonathan Coxhead, Leigh Taylor, Peter Leary, Megan S.R. Hasoon, Andrew M. Knight, Michelle D. Garrett, Ian Collins, Claire E. Eyers, Neil D. Perkins

**Affiliations:** 1 Newcastle University Biosciences Institute, Wolfson Childhood Cancer Research Centre, Newcastle University, Herschel Building, Level 6, Brewery Lane, Newcastle upon Tyne NE1 7RU, U.K.; 2 Centre for Proteome Research, Department of Biochemistry and Systems Biology, Institute of Systems, Molecular and Integrative Biology, University of Liverpool, Liverpool L69 7ZB, U.K.; 3 Newcastle University Clinical and Translational Research Institute, Preclinical In Vivo Imaging, Faculty of Medical Sciences, Newcastle University, Newcastle Upon Tyne NE2 4HH, U.K.; 4 Department of Molecular Physiology and Cell Signalling, Institute of Systems, Molecular and Integrative Biology, University of Liverpool, Liverpool L69 7ZB, U.K.; 5 Bioinformatics Support Unit, Faculty of Medical Sciences, Newcastle University, Newcastle Upon Tyne NE2 4HH, U.K.; 6 School of Biosciences, University of Kent, Stacey Building, Canterbury, Kent CT2 7NJ, U.K.; 7 Division of Cancer Therapeutics, The Institute of Cancer Research, Sutton SM2 5NG, U.K.

DNA replication stress resulting from the activity of oncogenes such as MYC, is a common feature of cancer cells. To cope with this challenge, tumours become addicted to ATR/CHK1 signalling, thus making these kinases attractive targets for anti-cancer therapies. Here, we demonstrate that the RelA(p65) NF-κB subunit is an important regulator of MYC induced DNA replication stress *in vivo*. Using the Eμ-Myc model of B-cell lymphoma, mice mutated at the putative CHK1 T505 phosphosite (T505A) in the RelA transactivation domain, exhibited reduced survival. Moreover, and in contrast with wild-type (WT) Eμ-Myc lymphomas, RelA T505A Eμ-Myc lymphomas are resistant to treatment with the CHK1 inhibitor CCT244747. Total protein and phosphopeptide proteomic analysis revealed that the response of RelA T505A Eμ-Myc lymphomas to a single acute dose of CCT244747 *in vivo*, was both reduced and different from WT cells. Subsequent examination of ATR/CHK1 signalling components revealed loss of expression of the ATR/CHK1 adaptor protein Claspin in RelA T505A Eμ-Myc lymphomas. Taken together our data reveal a critical role for RelA as a regulator of the DNA replication stress *in vivo*. We propose that by maintaining high Claspin levels, phosphorylation of the T505 site by CHK1 is required for efficient activation of CHK1 by ATR, thus driving the reliance on signalling through the ATR/CHK1 pathway required to survive high levels of DNA replication stress in cancer cells.

## Introduction

The NF-κB (nuclear factor-κ-light-chain-enhancer of activated B cells) transcription factor family members are important regulators of immune and inflammatory responses [1]. Furthermore, aberrantly active NF-κB is associated with many human diseases, including cancer [2–4]. While the pathways leading to nuclear translocation of NF-κB provide a primary level of regulation of its activity, NF-κB subunits are also subject to a wide range of post-translational modifications (PTMs). These have major effects on NF-κB subunit function, including control of nuclear translocation, induction of protein degradation, enhancement of DNA binding, as well as transcriptional effects such as stimulation or inhibition of co-activator/corepressor binding [5–9]. PTMs provide a mechanism to differentially regulate the transcriptional activity of NF-κB in response to diverse stimuli in different cell types [4,10].

The C-terminal transactivation domain of the RelA(p65) NF-κB subunit [11] contains many highly conserved known and putative phosphorylation sites [5]. It has been proposed that different patterns of phosphorylation can control the specificity of NF-κB target gene activation or repression [4,12,13], resulting in context-dependent functions of NF-κB. Understanding the mechanistic basis for these contrasting effects has important implications for the role of NF-κB in tumorigenesis and the response to cancer therapies [4]. Previously, the Perkins group has shown that in cell lines, Checkpoint kinase 1 (CHK1) can phosphorylate the RelA C-terminal transactivation domain at Thr505, resulting in inhibition of tumour-promoting characteristics of NF-κB, including resistance to apoptosis, autophagy and cell proliferation/migration [14–17]. However, the *in vivo* significance of these effects was not known. Therefore, to learn more about this we have created a knock-in mouse model in which the mouse equivalent of the human RelA Thr505 residue (Thr504) is mutated to Ala. For consistency with previous publications, we still refer to this as being a T505A mutation. We proposed that by inactivating specific mechanisms of regulation, targeted knock-in mutations of NF-κB subunits have the potential to reveal aspects of their behaviour not seen with knockouts, inhibition through RNA interference or expression of the inhibitor IκBα. We predicted, based on previous results, that the T505A mutation should lead to a form of RelA with enhanced tumour-promoting activity and consistent with this we observed earlier onset of more aggressive disease in the N-nitrosodiethylamine (DEN) chemical carcinogenesis model of hepatocellular carcinoma [18]. However, while this study revealed higher levels of proliferation and DNA damage in RelA T505A hepatocytes, mechanistic insights into how this came about were lacking.

We therefore decided to investigate the effect of the RelA T505A mutation in a second model of cancer, the Eμ-Myc mouse model of B-cell lymphoma [19]. Given our previous work linking phosphorylation of this site to regulation of NF-κB by CHK1 [14–17,20], we predicted that this would be particularly relevant in a cancer model driven by the oncogene c-Myc that induces high levels of DNA replication stress [21]. A role for RelA had been implied in Myc-driven lymphoma. Although shRNA depletion of RelA *in vivo* was found not to affect progression of established lymphomas, it did result in resistance to cyclophosphamide treatment due to an impaired induction of cell senescence [22]. Similarly, inhibition of NF-κB through expression of a degradation resistant form of IκBα, revealed a requirement for NF-κB activity for therapy-induced senescence in the Eμ-Myc model itself [23].

Here, we further characterise the RelA T505A mouse and determine the consequences of mutating RelA at Thr505 on the DNA replication stress response *in vitro* and *in vivo*. This manuscript is one of four reports that together reveal both the complexity of NF-κB regulation of DNA replication stress and CHK1 signalling, as well as how defects in these pathways can lead to CHK1 inhibitor (CHK1i) resistance. Related to this manuscript we show elsewhere, also using the Eμ-Myc model of B-cell lymphoma, that deletion of the c-Rel NF-κB subunit results in loss of CHK1 protein, at least in part due to down-regulation of the deubiquitinase USP1 [24]. There, we propose that an important component of CHK1i resistance is loss of CHK1 itself. This contrasts with the results described in this manuscript on the effects of T505A, where we find that although Eμ-Myc *RelA*^T505A^ mice also display resistance to CHK1 inhibition, they retain CHK1 protein. Using phosphoproteomics, we demonstrate that the response of Eμ-Myc *RelA*^T505A^ lymphomas to CHK1 inhibition *in vivo* is different from that seen in wild-type counterparts, with fewer and different targets being affected. Here, we propose that reduced levels of *CLSPN* (Claspin), a regulator of CHK1 activity, is an important component of this effect. In an accompanying paper we confirm that reduced levels of Claspin, using a *Clspn*^+/−^ knockout mouse, have numerous and unexpected physiological consequences [25].

The focus of this current manuscript and our report investigating resistance to CHK1 inhibition in the c-Rel null mouse model [24], are the mechanisms that lead to defects in CHK1 activity. This removal or alteration of the target of the CHK1i is an important component in the development of resistance but is not the only change these cells need to undergo. In the final paper in this series, we bring the Eμ-Myc *RelA*^T505A^ and *c-Rel*^−/−^ models together to consider how these lymphomas cope with these defects in CHK1 signalling, which in wild-type Eμ-Myc lymphomas is required for the survival [26]. We demonstrate that both models have up-regulated compensatory signalling pathways. Moreover, we show that Eμ-Myc *RelA*^T505A^ and *c-Rel*^−/−^ lymphomas, while resistant to CHK1 inhibition are now sensitive to targeting these bypass pathways [26]. These results have implications for how CHK1i resistance might arise in human patients and, importantly, suggest potential combination or second line therapies to overcome this.

## Results

### The T505A mutation results in increased NF-κB activity *in vivo*

**Figure 1.**
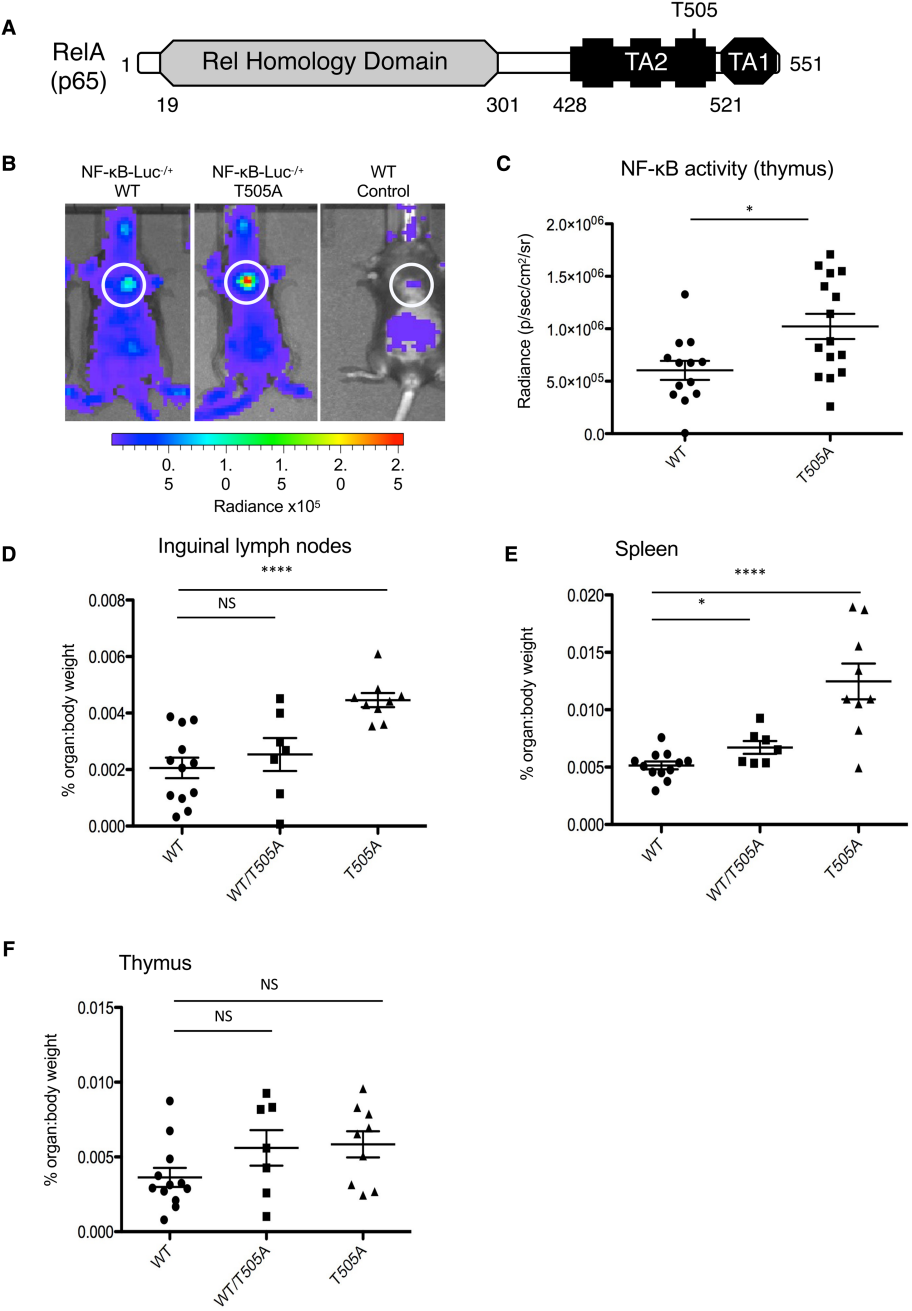
The phenotype of RelA T505A mice. (**A**) Schematic diagram of RelA showing the location of the Thr505 residue in Transactivation (TA) domain 2. (**B**) Representative images of *in vivo* NF-κB bioluminescence (radiance) in NF-κB-Luc^−/+^/WT, NF-κB-Luc^−/+^/T505A and NF-κB-Luc^+/+^/WT (WT control) mice. (**C**) Quantification of NF-κB bioluminescence (radiance) of thymic regions NF-κB-Luc^−/+^/WT and NF-κB-Luc^−/+^/T505A mice. *n* = NF-κB-Luc^−/+^/WT 12; NF-κB-Luc^−/+^/T505A 15; NF-κB-Luc^+/+^/WT 1. WT control bioluminescence was subtracted from Luc^−/+^ bioluminescence. (**D**–**F**) Percentage organ : body weight of (**D**) inguinal lymph nodes, (**E**) spleens and (**F**) thymi from WT, WT/T505A and T505A 12-week-old littermate mice. WT, *n* = 4 males, 8 females; WT/T505A *n* =^ ^2 males, 5 females; T505A *n* = 4 males, 5 females. Data represent mean ± SEM. *P *< 0.05. ****P < 0.0001. NS: not significant.

Before analysing the effect of the RelA T505A mutation on B-cell lymphoma progression in the Eμ-Myc model, we first characterised its effects in normal C57Bl/6 mice. Previous data from this laboratory in cell lines indicated that phosphorylation at the RelA Thr505 residue could inhibit NF-κB transcriptional activity and that mutation of this site to alanine removed this mechanism of negative regulation (Figure 1A) [14–18]. Therefore, to determine if a similar effect could be observed *in vivo*, RelA T505A mice were crossed on to transgenic NF-κB-Luc^−/+^ mice, which contain a reporter plasmid with three NF-κB binding sites driving expression of firefly luciferase. This enables real-time *in vivo* imaging and quantification of NF-κB activity as a bioluminescent signal [27]. NF-κB activity was measured and compared between healthy 12-week-old NF-κB-Luc^−/+^/wild-type and NF-κB-Luc^−/+^/T505A homozygous mice. As previously described [27], NF-κB activity could be seen in the thymus when live mice were imaged using the IVIS imaging system (Figure 1B,C). Significantly, this signal was amplified in the NF-κB-Luc^−/+^/T505A mice, directly demonstrating an *in vivo* transcriptional effect of this mutation.

### Phenotype of RelA T505A mice

Despite the lack of any overt immune phenotype, analysis of the secondary lymphoid organs of 12-week-old littermates revealed differences between wild-type, heterozygous and homozygous RelA T505A mutant mice. In particular, the inguinal lymph nodes and spleens were statistically significantly heavier (Figure 1D,E), but not visually larger (data not shown), in homozygous RelA T505A mice when compared with those from wild-type littermate controls. However, no differences in total cell numbers in these organs were observed (Supplementary Table S1). In addition, flow cytometric FACS analysis revealed that the percentages of B and T lymphocyte sub-populations, along with macrophage and dendritic cell populations, were similar in wild-type and T505A mice (Supplementary Table S2). Furthermore, despite the increase in NF-κB activity seen in the NF-κB-Luc^−/+^/T505A homozygous mice, no statistically significant differences were seen in the weight of the thymus (Figure 1F). A characteristic of the RelA T505A mice, although not one that we have attempted to quantify, is that they are more aggressive than wild-type counterparts. It is possible that the heavier spleens and inguinal lymph nodes in the RelA T505A mice is a consequence of a stress response in these mice sustained from minor injuries resulting from this aggressive nature.

### Mutation of RelA at T505 inhibits the pro-apoptotic effects of NF-κB following DNA replication stress

**Figure 2.**
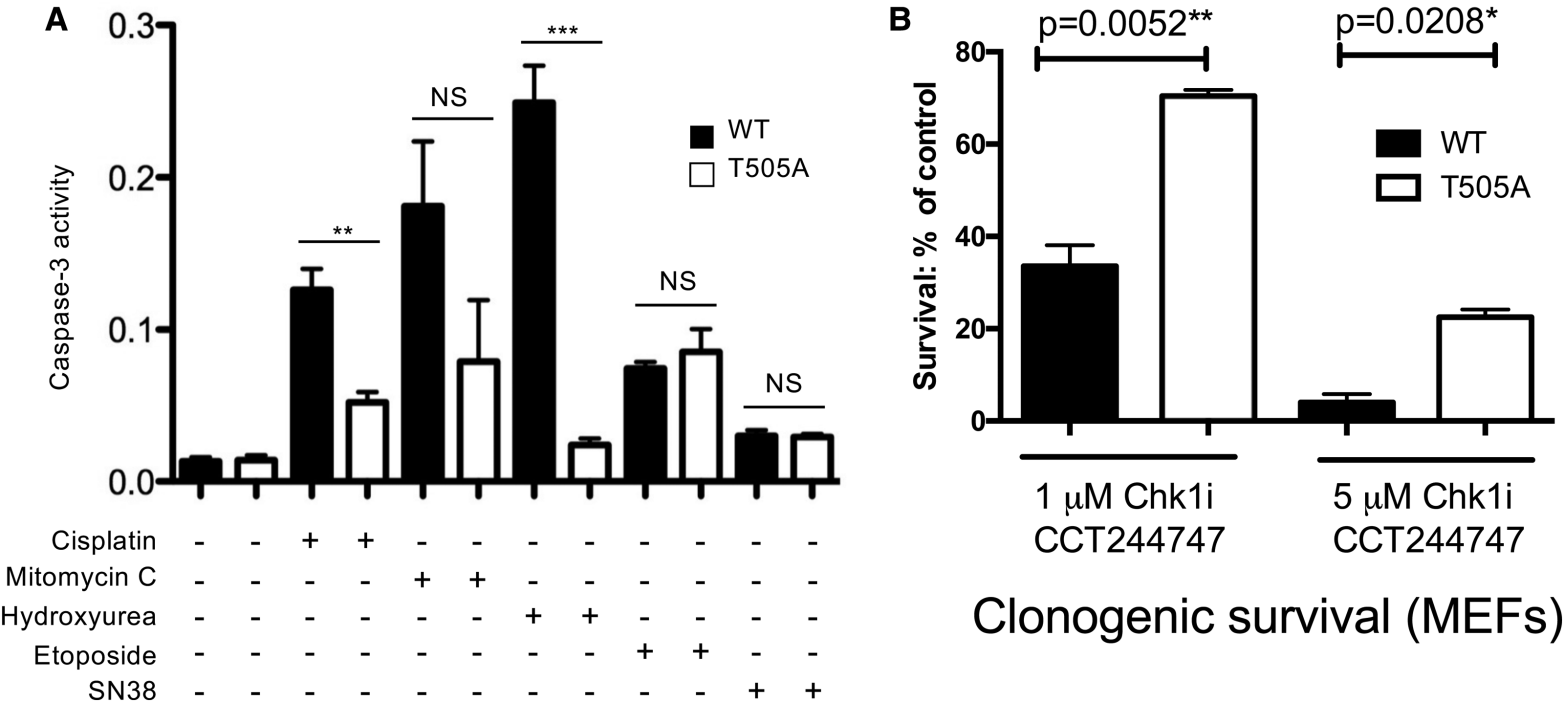
Mutation of RelA at T505 inhibits the pro-apoptotic effects of NF-κB following DNA replication stress. (**A**) *RelA*^T505A^ MEFs are resistant to apoptosis resulting from treatment with inducers of DNA replication stress. CASPASE 3 activity assay in immortalised WT and *RelA*^T505A^ MEFs after treatment with Cisplatin (4 μg/ml), Mitomycin C (1 μg/ml), Hydroxyurea (0.5 mM), Etoposide (15 μM) and the active metabolite of Camptothecin, SN38 (5 μM). All drugs were added for 16 h before analysis apart from SN38 (48 h). Results shown are the mean + SEM from three separate repeat experiments. (**B**) Increased clonogenic survival in *RelA*^T505A^ MEFs following CHK1 inhibitor treatment Clonogenic survival in WT and *RelA*^T505A^ MEFs following either treatment with either 1 μM (**P = 0.0052, Unpaired Student's *t*-test) or 5 μM (*P = 0.0208, Unpaired Student's *t*-test) of the CHK1 inhibitor, CCT244747 for 24 h.

RelA T505 phosphorylation has been shown to induce a pro-apoptotic form of NF-κB following stimulation with the chemotherapeutic drug and DNA cross-linker cisplatin [15,16]. However, these experiments were based on exogenous expression of wild-type (WT) and T505A RelA mutants in either human cell lines or *Rela*^−/−^ immortalised mouse embryonic fibroblasts (MEFs). Therefore, to confirm the role of endogenous RelA T505 phosphorylation as a regulator of the NF-κB response to DNA damage in this cell type we generated immortalised MEFs from *RelA*^T505A^ mice and wild-type littermates. Importantly, and in agreement with the previous data, mutation of endogenous RelA at T505 strongly protected cells from apoptosis induced by cisplatin and other inducers of DNA replication stress, the DNA cross-linker mitomycin C and hydroxyurea, an inhibitor of ribonucleotide reductase (Figure 2A, Supplementary Figure S1A,B). In contrast, there was no significant difference in apoptosis between WT and *RelA*^T505A^ MEFs when treated with etoposide, a Topoisomerase II inhibitor, or with SN38, a Topoisomerase I inhibitor and the active metabolite of camptothecin (Figure 2A, Supplementary Figure S1A). Western blot analysis revealed no significant effects on the other NF-κB subunits in the *RelA*^T505A^ MEFs (Supplementary Figure S1C). We had also previously observed that *Rela*^−/−^ MEFs reconstituted with RelA T505A underwent significant remodelling to their actin cytoskeleton [16]. Interestingly, a similar phenotype was observed in the *RelA*^T505A^ MEFs, with cells appearing larger and displaying a more intense actin staining (Supplementary Figure S1D).

Since the RelA T505 residue can be phosphorylated by the checkpoint kinase CHK1 [17], we next determined whether the RelA T505A mutation would also affect cell survival in response to treatment with CHK1 inhibitors, an important class of novel cancer therapeutics currently in clinical trials [28]. Interestingly, in both cell viability and clonogenic survival assays the *RelA*^T505A^ MEFs were significantly more resistant to the CHK1i CCT244747 (Figure 2B, Supplementary Figure S2A) [29], and the structurally unrelated CHK1i MK8776 (Supplementary Figure S2B) [30,31] compared with matched WTs.

### Mutation of RelA Thr505 results in reduced survival of Eμ-Myc/*Rela^T505A^* mice and resistance to CHK1 inhibitors

**Figure 3.**
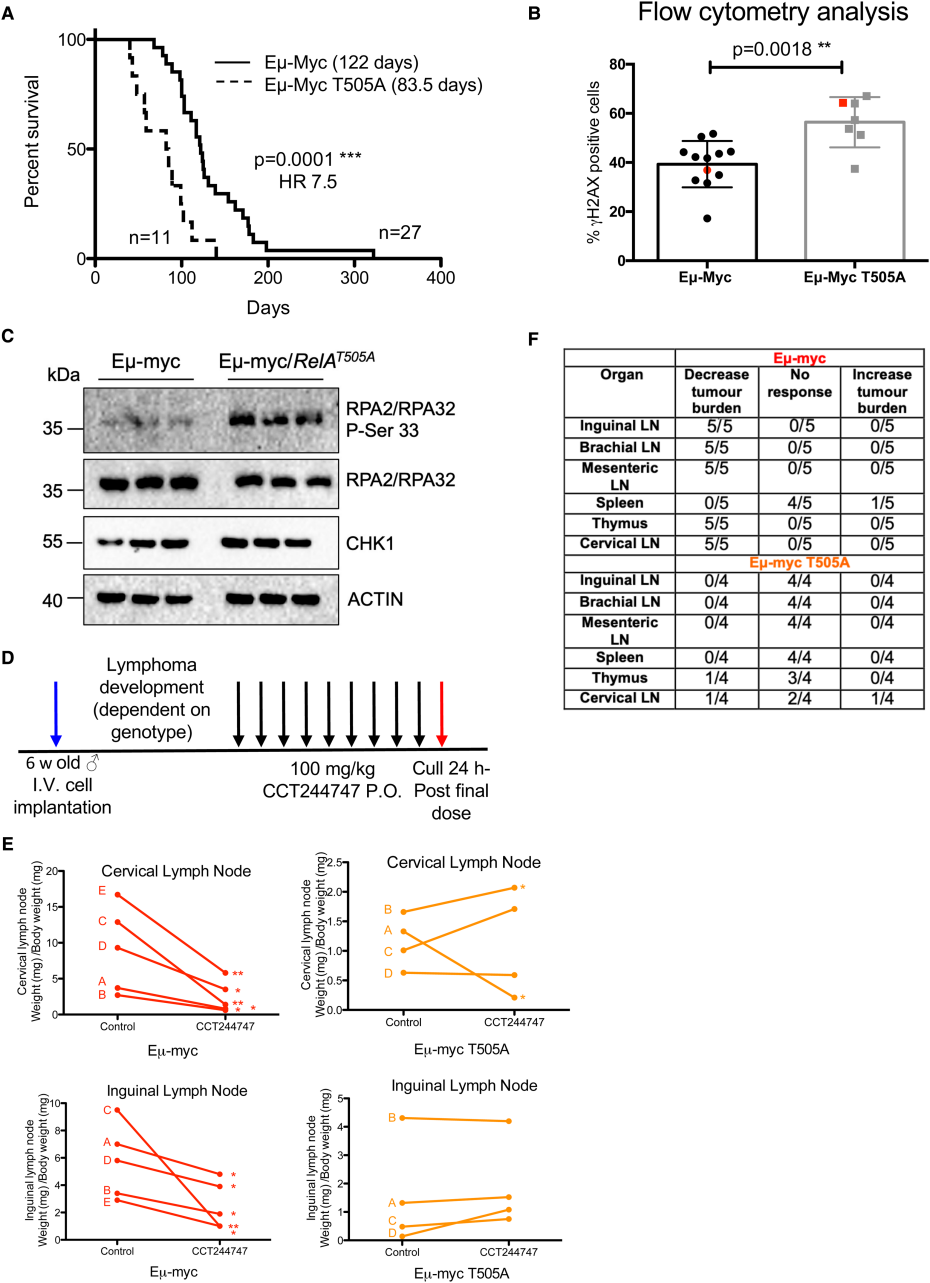
The RelA T505A mutation leads to reduced survival of Eμ-Myc*/Rela^T505A^* mice, genomic instability and resistance to CHK1 inhibitors. (**A**) Reduced survival of Eμ-Myc*/Rela^T505A^* mice. Kaplan–Meier survival curves for Eμ-Myc (*n* = 27) and homozygous Eμ-Myc*/Rela^T505A^* male mice (*n* = 11). Overall survival is significantly shorter in Eμ-Myc/*RelA*^T505A^ mice (***P = 0.0001, Mantel–Cox test) and hazard ratio (HR) analysis indicates that these mice are at 7.5 times greater risk of dying earlier due to lymphoma compared with Eμ-Myc mice. The median survival for each genotype is indicated. (**B**) Increased genomic instability in lymphomas from Eμ-Myc*/Rela^T505A^* mice. Flow cytometric analysis of B cells prepared from Eμ-Myc and Eμ-Myc*/Rela^T505A^* spleens and stained for γH2AX, a marker of DNA damage (**P = 0.0018, Unpaired Student's *t*-test). The red dot in each case illustrates the mouse shown in Supplementary Figure S3A,B. (**C**) Western blot analysis of RPA2 phospho-Ser33, total RPA2, total CHK1 and ACTIN in snap frozen tumour extracts prepared from three different reimplanted WT Eμ-Myc and Eμ-Myc*/Rela^T505A^* tumours mouse inguinal lymph nodes. (**D**) Schematic diagram illustrating the CHK1i *in vivo* study in Eμ-Myc and Eμ-Myc*/Rela^T505A^* mice. Six-week-old C57Bl/6 WT mice were implanted with either Eμ-Myc or Eμ-Myc*/Rela^T505A^* lymphoma cells (blue arrow) and once tumours became palpable were treated with either 100 mg/kg CCT244747 p.o or vehicle control once daily for 9 days (black arrows). Mice were euthanised 24 h after the final dose (red arrow) and tumour burden assessed. (**E**) Reimplanted Eμ-Myc/*Rela^T505A^* lymphoma cells are resistant to CHK1 inhibition *in vivo.* Line graphs showing the mean response of the five reimplanted Eμ-Myc (red) and four Eμ-Myc/*Rela^T505A^* (orange) tumours and their response to CCT244747. A response was defined as a significant reduction (or increase) in tumour burden (*P* < 0.05) using Unpaired Student's *t*-tests (*P < 0.05, **P < 0.01). (**F**) Table showing the response of reimplanted Eμ-My and Eμ-Myc/*Rela^T505A^* tumours to CCT244747, in all sites where lymphoid tumour burden is anticipated in this model.

These data confirmed and extended the original observation that RelA T505 phosphorylation is an important regulator of the cellular response to inducers of DNA replication stress. Therefore, since overexpression of MYC is a feature of many types of cancer and results in DNA replication stress leading to genomic instability and tumorigenesis [32,33], we next investigated the effect of the RelA T505A mutation in the well-established Eμ-Myc mouse model of B-cell lymphoma [19]. Our prediction, based on our previous data, was that RelA T505A mice would exhibit reduced survival times in this model of lymphomagenesis. Indeed, homozygous Eμ-Myc*/Rela^T505A^*mice had a significantly shorter overall survival (median survival 83.5 days) when compared with the Eμ-Myc mice (median survival 122 days) (Figure 3A). Moreover, consistent with RelA T505 phosphorylation being a regulator of the response to DNA replication stress, we observed significantly higher levels of γH2AX, a marker of DNA damage and genomic instability in Eμ-Myc*/Rela^T505A^* lymphomas (Figure 3B, Supplementary Figure S3A,B). We also observed high levels of phosphorylation at Ser 33 of replication protein A (RPA) 2 (RPA 32), a marker for ATR activation and DNA replication stress (Figure 3C). RPA is a eukaryotic ssDNA-binding protein that is essential for DNA replication and repair [34]. It is not only crucial for the recruitment and activation of ATR, it is also an ATR target [35,36]. In response to genotoxic stress, RPA32 is phosphorylated on S33 by ATR and this phosphorylation subsequently stimulates phosphorylation by Cyclin-CDKs and DNA-PK to yield hyperphosphorylated RPA [37]. RPA2 is also a target for ATM and DNAPK [38]. Together these results suggested that Eμ-Myc*/Rela^T505A^* lymphomas are undergoing higher levels of genomic instability than their WT equivalents. Even though these results suggested a defect in checkpoint kinase signalling, CHK1 protein levels were similar between WT Eμ-Myc and Eμ-Myc*/Rela^T505A^* lymphomas (Figure 3C). Furthermore, western blot analysis revealed no significant effects on the other NF-κB subunits apart from increased levels of RelB in T505A cells (Supplementary Figure S3C).

Given that *RelA*^T505A^ MEFs displayed resistance to CHK1i treatment (Figure 2B, Supplementary Figure S2A,B), we hypothesised that Eμ-Myc/*Rela*^T505A^ tumours would also show altered sensitivity to CHK1 inhibition *in vivo*. To investigate, we used the CHK1i CCT244747 as it has shown efficacy in a transgenic model of MYCN-driven neuroblastoma [29]. Moreover, we have previously shown that the related CHK1i CCT245737 (SRA737) inhibits the growth of reimplanted WT Eμ-Myc cells [39]. To perform this analysis, we used a tumour reimplantation model, where wild-type C57Bl/6 mice receive lymphoma cells from Eμ-Myc mice via tail vain injection, with development of tumours and their location being monitored over time. While performing initial characterisation of the model, we observed that as expected, all of the WT Eμ-Myc cells ‘homed’ to organs of lymphoid origin; the spleen, lymph nodes and thymus (Supplementary Figure S3D), as we have also seen previously with Eμ-Myc lymphomas from c-Rel null mice [40]. This was not the case with approximately half of the reimplanted Eμ-Myc*/Rela^T505A^* tumours. Strikingly, we found extensive B-cell tumour burden in the livers of four of the eight Eμ-Myc*/Rela^T505A^* tumours, and within the lungs in two of the eight lymphomas used in this experiment (Supplementary Figure S3D). These mice also presented with some tumour burden that was apparent in at least one lymphoid organ, such as within the lymph nodes within the mesentery. However, a quarter of the mice reimplanted with Eμ-Myc*/Rela^T505A^* cells failed to exhibit any obvious site of tumourigenesis, despite reduced food and water consumption which acts as a robust biomarker for disease in this model [41]. These observations were consistent, with the same lymphoma samples exhibiting the same tumour homing characteristics following different reimplantations. Although this result was of great interest, since it implies that RelA T505 phosphorylation is an important mediator of cell invasiveness, motility and metastatic potential *in vivo*, for the purposes of this study we focussed on those lymphoma samples whose lymph node homing behaviour mimicked that of wild-type Eμ-Myc samples. We did not detect this phenotype or any other clinical differences in the presentation of the primary lymphomas in the Eμ-Myc/*Rela*^T505A^ mice. However, this may result from these mice being culled at pre-determined endpoints (see Methods), before visible metastatic tumour growth in the non-lymphoid organs can be detected.

The effectiveness of CCT244747 *in vivo* was determined by analysing its effect on the growth of reimplanted WT Eμ-Myc, and Eμ-Myc/*Rela*^T505A^ tumours. Each tumour was implanted into six syngeneic C57Bl/6 recipient mice: three were treated orally with CCT244747 once a day for 9 days (Supplementary Figure S4A), while three received a vehicle control (Supplementary Figure S3D). After treatment, we observed a striking reduction in lymphoid tumour burden in all mice reimplanted with WT Eμ-Myc tumours (Figure 3E,F, Supplementary Figure S4B). In contrast, three of the four Eμ-Myc/*Rela*^T505A^ tumours showed no significant reduction in lymphoid tumour burden after CCT244747 treatment, while the remaining tumour exhibited only a partial response with a reduction in tumours of the thymus and cervical lymph nodes. These data confirmed that the NF-κB mutant Eμ-Myc tumours have disrupted checkpoint kinase signalling pathway, bypassing a requirement for CHK1 activation, thus rendering them insensitive to the CHK1i.

### RNA Seq and proteomic analysis of WT and Rela T505A Eμ-Myc lymphomas

**Figure 4.**
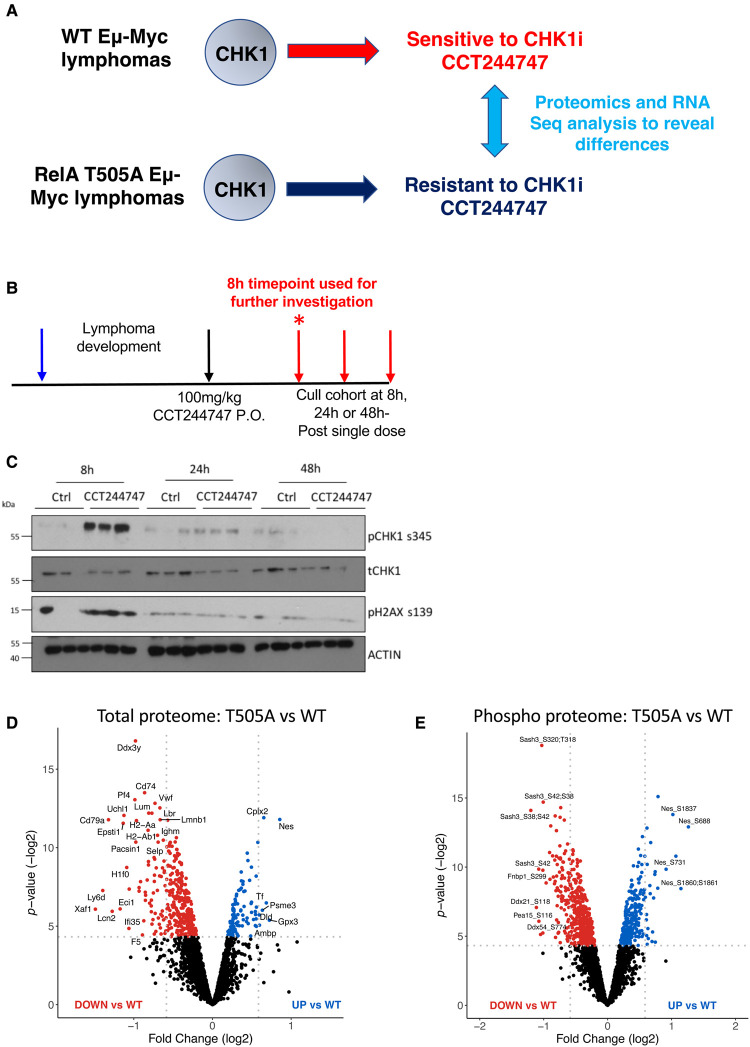
CCT244747 efficiently targets the CHK1 kinase, eliciting a strong effect in WT but not RelA T505A mutated lymphomas. (**A**) Diagram depicting the strategy of using RNA Seq and proteomics analysis to reveal the changes in gene and protein expression that lead to CCT244747 resistance in RelA T505A Eμ-Myc/*Rela^T505A^* lymphomas. (**B**) Schematic diagram illustrating the single dose CHK1i *in vivo* study in Eμ-Myc and Eμ-Myc*/Rela^T505A^*mice. Six-week-old C57Bl/6 WT mice were implanted with either Eμ-Myc or Eμ-Myc*/Rela^T505A^* lymphoma cells (blue arrow) and once tumours became palpable were treated with either a single dose of 100 mg/kg CCT244747 p.o or vehicle control (black arrows), and then mice were then euthanised at either 8 h, 24 h or 48 h later (red arrow). The star (*) denotes the 8-h timepoint which was taken forward for use in future RNA-Seq and proteomic studies. (**C**) Western blot analysis of phospho-Ser345 CHK1, CHK1, phospho-S139 H2AX (yH2AX) or ACTIN in snap frozen tumour extracts prepared from reimplanted Eμ-Myc tumours mouse inguinal lymph nodes at given timepoints (8 h, 24 h or 48 h) following a single dose of CCT244747. Please note that these samples are further reimplantations of the same tumours analysed in the chronic dosing study of CCT244747 (Figure 3E**,**F). (**D,E**) Volcano plot demonstrating the significant number of total protein (**D**) and phospho proteome (**E**) differences between the Eμ-Myc WT and Eμ-Myc*/Rela^T505A^* lymphomas. Down-regulated proteins are shown with red dots and up-regulated proteins are shown with blue dots.

This resistance to CHK1i treatment, and our previous data suggesting RelA T505 is a potential CHK1 phosphosite [14–17] implied that ATR/CHK1 signalling might be compromised in the Eμ-Myc/*Rela*^T505A^ cells. We therefore wanted to explore how these cells respond at an early time point to a single dose of CCT244747 *in vivo* using combined RNA Seq and proteomic analysis (Figure 4A, Supplementary Figure S4C and Supplementary Data Files S1–S3). By examining this acute response, we reasoned that we could gain insights into how signalling in these cells had been rewired, something not possible with longer CCT244747 treatment where the mixture of dead, dying and surviving lymphoma cells was likely to confound analysis. To determine the optimum timepoint to study these changes, mice reimplanted with WT Eμ-Myc lymphomas were culled at various timepoints following a single dose of CCT244747 (Figure 4B). We observed a significant DNA damage response, as measured by an increase in phosphorylation of H2AX at Ser 139, after 8 h of treatment that diminished at 24 and 48 h (Figure 4C). Moreover, we observed high levels of CHK1 phosphorylation at Ser 345 at the 8-h time point, an ATR-mediated phosphorylation site that typically becomes hyperphosphorylated upon CHK1 inhibition [42] 8 h after treatment that diminished at 24 and 48 h (Figure 4C). Hence, we used this 8-h timepoint in all further studies.

The RNA Seq analysis revealed that the RelA T505A mutation has a widespread and significant effect on RNA expression in Eμ-Myc lymphomas without CHK1i treatment, with 1139 transcripts showing significant (adj*P* < 0.05) differences (Supplementary Data File S4). Of these, analysis using the Gprofiler website (https://biit.cs.ut.ee/gprofiler/gost) to search the TRANSFAC database of transcription factor binding sites identified that 218 (19%) are potential RelA targets (Supplementary Data File S4). These were approximately evenly distributed between up and down-regulated genes (Supplementary Figure S5A). This gene list includes many other transcriptional regulators, suggesting that many effects on gene expression are secondary to direct effects on NF-κB regulated genes. EnrichGO analysis of putative RelA regulated genes and down-regulated mRNAs in RelA T505A cells did not reveal any functional groups with a *q*-value < 0.05. In our parallel report examining up-regulated compensatory bypass pathways in CCT244747 resistant Eμ-Myc lymphomas we examine in more detail the up-regulated mRNAs in RelA T505A cells [26].

To explore regulation of the proteome in WT and RelA T505A reimplanted lymphomas, with and without 8 h treatment with CCT244747, we used tandem mass tag (TMT)-based isobaric labelling to quantify relative changes in both total protein levels and phosphopeptide abundance (Supplementary Figure S4C). Of the ∼4000 proteins identified at a 1% false discovery rate (FDR), ∼2500 were quantified in at least three biological replicates (Supplementary Data File S1). At the phosphopeptide level, we identified over 6500 phosphopeptides, quantifying ∼3350 in at least three replicates (>4500 in at least two bioreps; Supplementary Data File S1). These contained 3193 unique phosphosites with a *ptm*RS >=0.998 equivalent to a FLR of 1% based on [43]. This data revealed many proteomic differences between WT and RelA T505A reimplanted lymphomas before CCT244747 treatment (Figure 4D,E). Functional annotation analysis of these changes using DAVID (https://david.ncifcrf.gov/) revealed many significantly affected categories with a Benjamini *q*-value <0.05 (Supplementary Data File S5). These included mRNA splicing, ubiquitin conjugation, cell adhesion and cytoskeletal networks. Only 137/380 proteins with phosphopeptide differences between WT and RelA T505A lymphomas also displayed altered protein expression (Supplementary Figure S7B and Supplementary Data File S6). There was surprisingly little overlap between changes in RNA expression and changes in the total proteome, with only 46/469 proteome changes (*P* < 0.05) showing a significant change at the mRNA level (Supplementary Figure S5B and Supplementary Data File S6). This indicates that many phenotypic changes in the Eμ-Myc/*Rela*^T505A^ lymphomas are likely mediated by post-transcriptional mechanisms, such as regulation of ubiquitin mediated proteolysis. Although the functional annotation analysis above reveals some pathways that may play a role in the altered phenotype of Eμ-Myc/*Rela*^T505A^ lymphomas described above, none of them obviously provided an explanation for the CCT244747 resistance we observe.

### Eμ-Myc Rela T505A lymphomas exhibit an altered response to CHK1 inhibition

**Figure 5.**
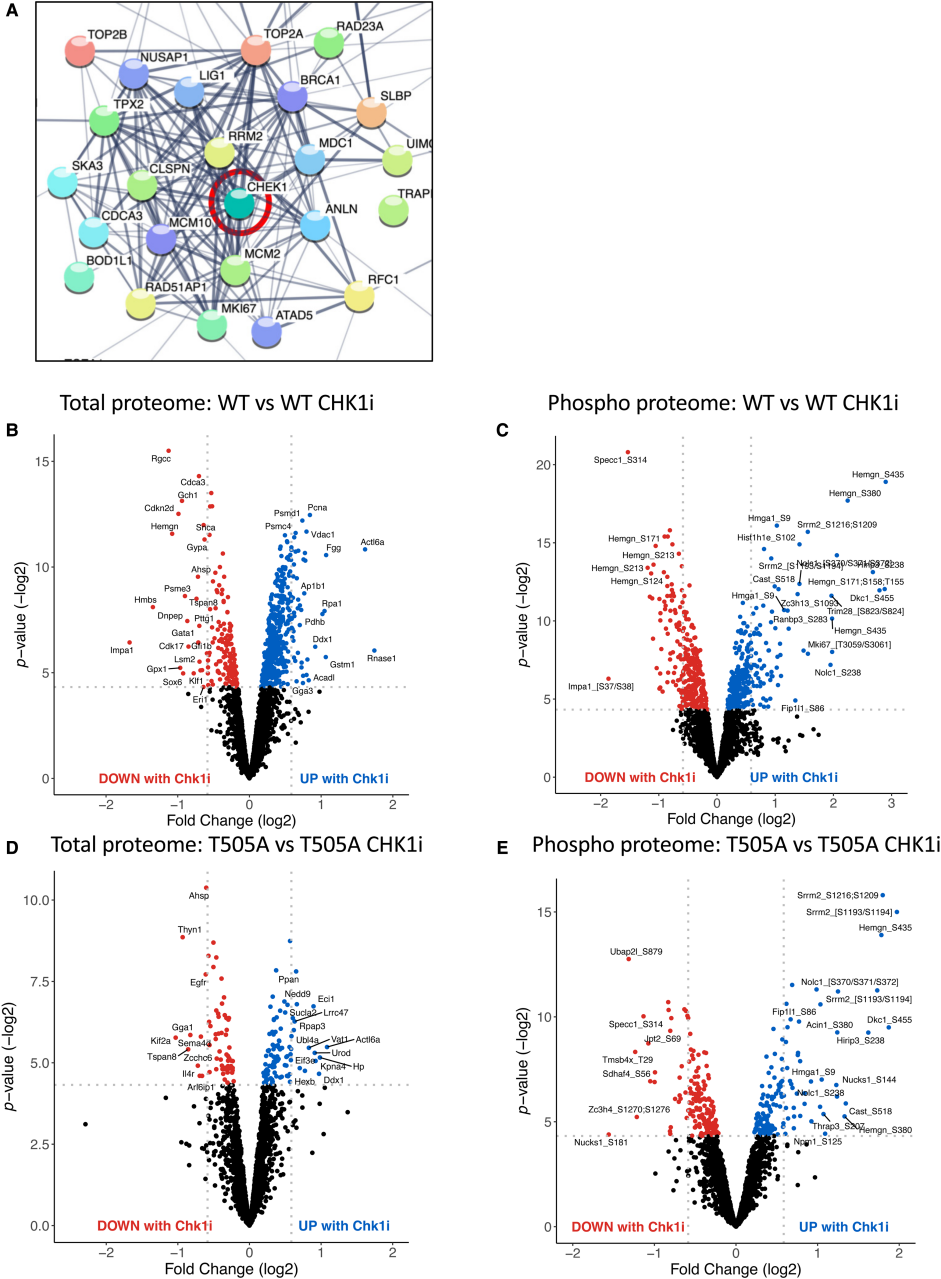
CCT244747 efficiently targets the CHK1 kinase. (**A**) STRING analysis showing that a cluster of proteins associated with CHK1 had down-regulated phosphorylation was upon CCT244747 treatment in Eμ-Myc WT tumours after a single dose of the CHK1i. (**B,C**) Volcano plots demonstrating a significant number of CCT244747 effects in Eμ-Myc WT lymphomas on both the total (**B**) and phospho (**C**) proteome, with both down- (red dots) and up-regulation (blue dots) being observed. (**D**,**E**) Volcano plots demonstrating a significant number of CCT244747 effects in Eμ-Myc T505A lymphomas on both the total (**D**) and phospho (**E**) proteome, with both down- (red dots) and up-regulation (blue dots) being observed. Please note that figures (**B**,**C**), showing data from WT Eμ-Myc lymphomas are replicated in another manuscript [24], where they are used for comparison with data from Eμ-Myc*/cRel*^−/−^ lymphomas.

STRING analysis (https://string-db.org/) of the phosphoproteomic data from WT Eμ-Myc lymphomas revealed a cluster of proteins known to be associated with CHK1, whose phosphorylation was reduced upon CCT244747 treatment (Figure 5A, Supplementary Figure S6 and Supplementary Data File S7). This data confirmed effective targeting of CHK1 by CCT244747 *in vivo*. In Figure 5A CHK1 has been manually added to the analysis to directly show the links while in Supplementary Figure S6, the cluster of phosphoproteins in shown without addition of CHK1.

Further analysis of this data demonstrated a significant number of CCT244747 effects in WT Eμ-Myc lymphomas, with 622 proteins and 625 phosphopeptides exhibiting statistically significantly up- or down-regulation (*P* ≤ 0.05) (Figure 5B,C, Supplementary Data File S1). In contrast, relatively few effects of CHK1 inhibition were seen at the mRNA level from parallel RNA Seq data analysis (Supplementary Figure S7A and Supplementary Data Files S2 and S3). This indicated that 8 h after a single dose of CCT244747, the vast majority of the effects seen are post-transcriptional in nature. Strikingly, comparatively few significant changes relative to WT cells were seen on the total and phosphoproteomes following acute CCT244747 treatment of RelA T505A Eμ-Myc lymphomas, with only 157 proteins and 315 phosphopeptides being differentially regulated (*P* ≤ 0.05) (Figure 5D,E, Supplementary Data File S1). Please note that the data from WT Eμ-Myc mice shown here is also used in our study on cRel^−/−^ Eμ-Myc lymphomas [24]. These experiments were performed in parallel as part of the same larger study.

**Figure 6.**
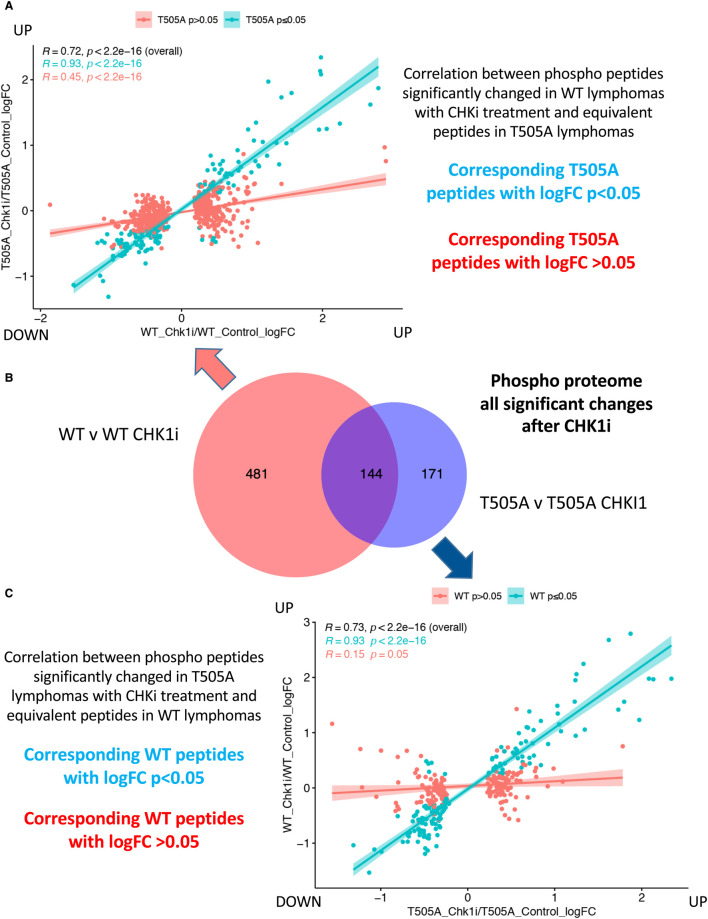
Eμ-Myc WT lymphomas show a strong response to CHK1 inhibition via changes in the phospho peptide landscape. (**A**) Correlation between phosphopeptides significantly changed in Eμ-Myc WT lymphomas with CHKi treatment and equivalent peptides in Eμ-Myc*/Rela^T505A^* lymphomas. (**B**) Venn diagram illustrating that of the 625 phosphopeptide changes observed in Eμ-Myc WT tumours following acute CCT244747 treatment, 144 were also changed in Eμ-Myc*/Rela^T505A^* lymphoma cells following inhibitor treatment (site localisation not considered). (**C**) Correlation between phosphopeptides significantly changed in Eμ-Myc*/Rela^T505A^* lymphomas with CHKi treatment and equivalent peptides in WT lymphomas.

**Figure 7.**
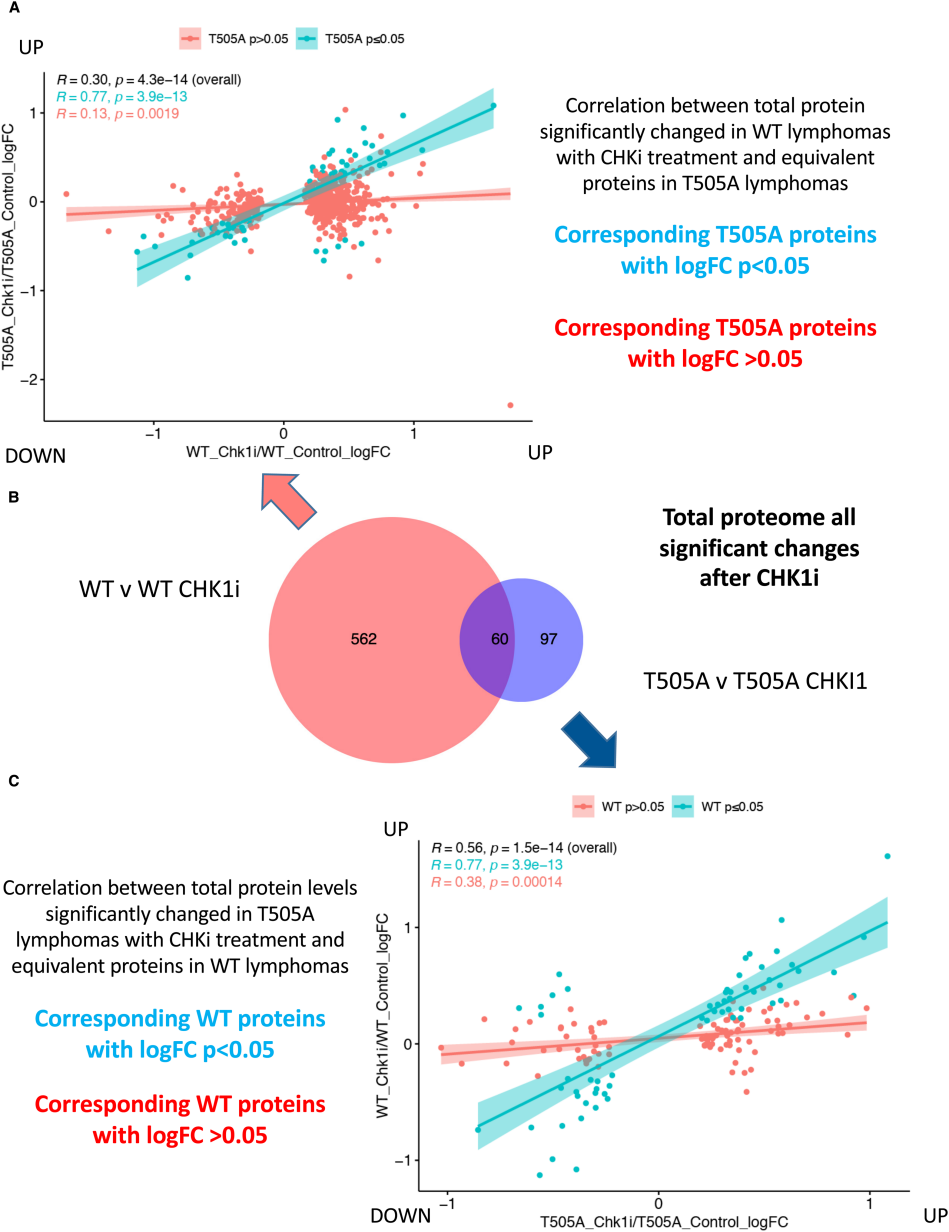
RelA T505A lymphomas exhibit few changes in total protein levels following CHK1 inhibition. (**A**) Correlation between total proteins significantly changed in WT lymphomas with CHKi treatment and equivalent proteins in Eμ-Myc*/Rela^T505A^* lymphomas. (**B**) Venn diagram illustrating that of the 662 total protein changes observed in Eμ-Myc WT tumours following acute CCT244747 treatment, only 60 were also changed in Eμ-Myc*/Rela^T505A^* lymphoma cells following inhibitor treatment. (**C**) Correlation between total protein levels significantly changed in Eμ-Myc*/Rela^T505A^* lymphomas with CHKi treatment and equivalent proteins in WT lymphomas.

When these CCT244747-induced phosphopeptide and protein differences between the WT Eμ-Myc and Eμ-Myc*/Rela^T505A^* cells were analysed in more detail, we observed that less than half were common between WT and RelA T505A lymphoma cells (Figures 6 and 7). Detailed analysis of these differences revealed that of the phospho peptides and proteins significantly changed in WT cells, relatively few show significant changes in RelA T505A cells (Figures 6A and 7A). Similarly, of the phospho peptides and proteins significantly altered in RelA T505A cells upon treatment with CCT244747, over half were not observed following treatment in WT cells (Figures 6C and 7C). These phosphorylation differences could not be completely explained by the changes in the total proteome between WT and RelA T505A lymphoma cells. Only 37/135 of the proteins with phosphopeptide changes unique to RelA T505A cells had altered protein expression between WT and RelA T505A lymphomas (Supplementary Figure S7B and Supplementary Data File S6). Conversely only 66/297 of the proteins with phosphopeptide changes unique to WT cells had altered protein expression between WT and RelA T505A lymphomas (Supplementary Figure S7C and Supplementary Data File S6).

String analysis (https://string-db.org/) was then used to identify genes and proteins with altered expression in the RNA Seq and total proteome analysis from Eμ-Myc*/Rela^T505A^* and wild-type Eμ-Myc lymphomas (no CCT244747 treatment) that had known links to CHK1. This revealed 24 mRNAs and 16 proteins, respectively; further analysis using Biogrid (https://thebiogrid.org/) identified 5, Mdm3, Casp8, Rad 51, Top2a and Tp53bp1, reported to bind directly to CHK1 (Supplementary Figure S7D and Supplementary Data File S8). A further 7 proteins, Pik3c3, Parp2, Nek7, Ddb2, Mcm9 and 10 and Gm2423 (Ywhaq) were related to CHK1 interacting proteins (Supplementary Figure S7D and Supplementary Data File S8). Changes in the expression of these candidate proteins, therefore, has the potential to affect CHK1 activity and substrate targeting. However, further experimentation would be required to evaluate whether this is explains the differential response to CCT244747 treatment seen between WT Eμ-Myc and Eμ-Myc*/Rela^T505A^* lymphomas.

We, therefore, conclude that the nature of this CCT244747 response is profoundly different between these two systems, with the changes in RelA T505A lymphoma cells being both smaller in number and different from those in WT lymphoma cells. This was consistent with the lack of effectiveness on lymphoma growth seen with long term CCT244747 dosing (Figure 3D–F).

### Claspin mRNA levels predict overall survival in Eμ-Myc B-cell lymphoma

**Figure 8.**
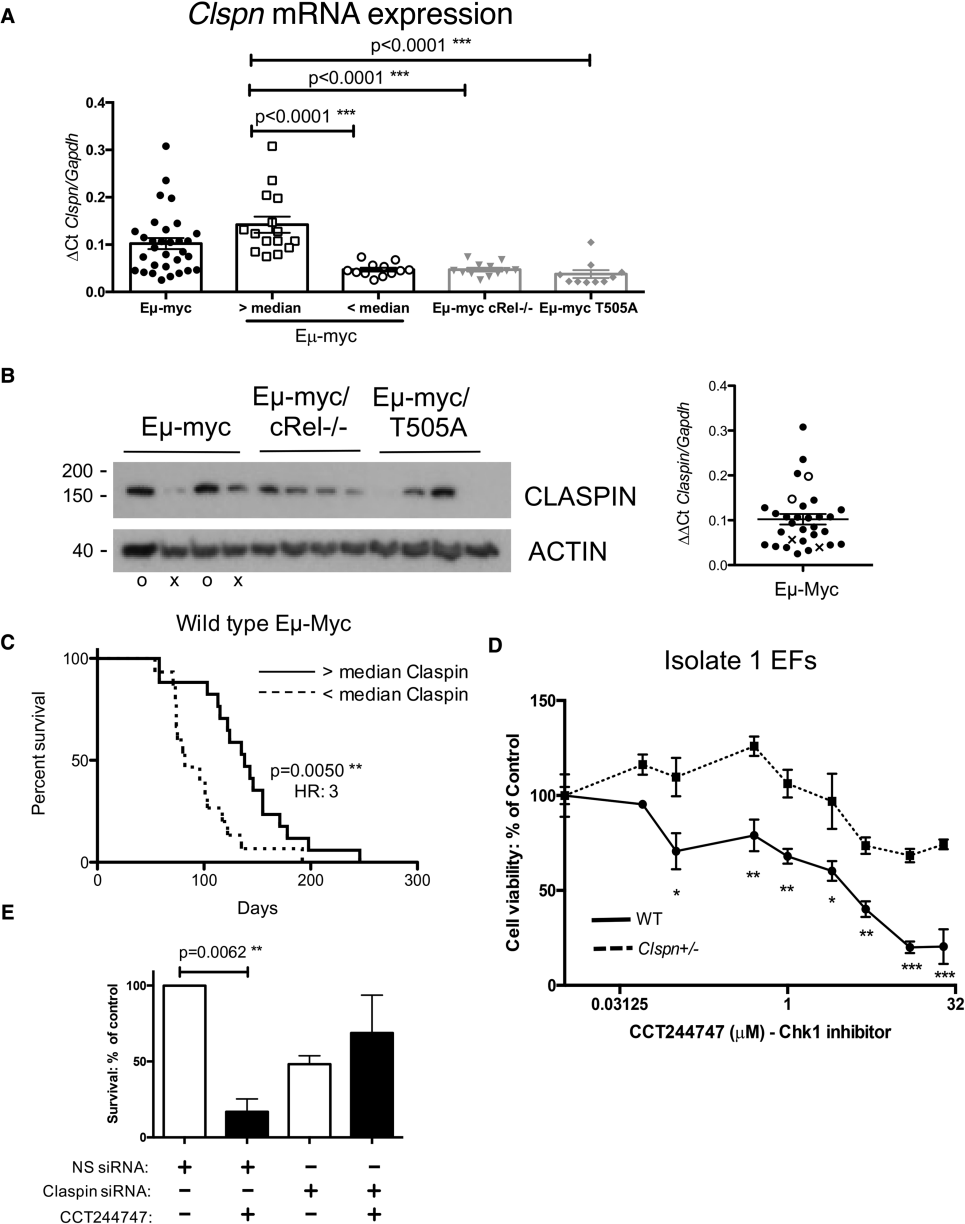
Eμ-Myc/*RelA*^T505A^ lymphomas fail to induce Claspin expression. (**A**) Reduced Clspn mRNA expression in Eμ-Myc/*c-rel*^−/−^ and Eμ-Myc/*Rela^T505A^* lymphomas. qRT-PCR data showing relative *Clspn* mRNA expression in tumorigenic spleens from Eμ-Myc (*n* = 30), in Eμ-Myc/*c-rel*^−/−^ (*n* = 12) and Eμ-Myc/*Rela^T505A^* (*n* = 8) mice. Data represent mean ± SEM. **P < 0.01 (Unpaired Student's *t*-test), each point is an individual mouse. (**B**) Claspin protein expression is also reduced in Eμ-Myc*/c-Rel*^−/−^ and Eμ-Myc/*Rela^T505A^* lymphomas. Western blot analysis of Claspin using extracts prepared from Eμ-Myc, Eμ-Myc*/c-Rel*^−/−^ or Eμ-Myc/*Rela^T505A^* tumorigenic spleens. Accompanying scatter plot illustrates which wild-type mice with either high (o) or low (x) *Clspn* mRNA expression were used for protein analysis. (**C**) WT Eμ-Myc mice with lower Claspin levels develop lymphoma earlier. Kaplan–Meier survival analysis of WT Eμ-Myc mice comparing above and below median level expression of *Clspn* mRNA. Please note that this figure is also used in a related manuscript [25]. (**D**) Primary fibroblasts from *Clspn*^+/−^ mice [25] are resistant to Chk1 inhibition. Cell viability (Prestoblue assay) in wild-type and Clspn^+/−^ primary ear fibroblasts following treatment with increasing concentrations of the Chk1 inhibitor, CCT244747. *P < 0.05, **P < 0.01, ***P < 0.001 (One-way ANOVA with Sidak *post hoc* test). (**E**) siRNA knockdown of CLSPN leads to CCT244747 resistance. U2OS cells were transfected with siRNA targeting CLSPN or a non-specific (NS) siRNA control. 48 h post-transfection, cells were treated for 24 h with 1 mM CHK1 inhibitor, CCT244747, or solvent controls before re-seeding onto 6-well plates. Colonies were fixed 21 days later and counted.

The difference in the (phospho)proteome response to inhibitor treatment implied that the RelA T505A mutation was inducing a change or defect in ATR/CHK1 signalling in response to DNA replication stress in Eμ-Myc lymphoma cells. Therefore, in addition to the RNA Seq and proteomic analysis, we took a candidate approach and used qPCR to analyse the mRNA expression of core components of ATR/CHK1 signalling, *Atr*, *Atrip*, *Clspn*, *Topbp1*, *Rad17* and *Chek1* (Supplementary Figure S8A–C). By contrast with the RNA Seq and proteomic analysis using reimplanted lymphoma cells, this was performed using primary lymphoma spleen tissue from WT Eμ-Myc*/*and Eμ-Myc*/Rela^T505A^* mice. In addition, since the NF-κB subunit, c-Rel has previously been shown to induce the expression of the ATR–CHK1 regulator Claspin in cell lines [44], we also included samples from primary c-Rel^−/−^ Eμ-Myc lymphomas [40] in this analysis as a control. Interestingly, this revealed significantly lower *Clspn* mRNA expression in Eμ-Myc/*Rela*^T505A^ and Eμ-Myc/*cRel*^−/−^ mice relative to their wild-type littermates (Figure 8A). In contrast, no effects were seen on *Atr*, *Atrip*, *Topbp1*, *Rad17* and *Chek1* mRNA levels (Supplementary Figure S8B), with western blot analysis confirming no changes to RAD17, ATRIP and ATR protein levels between WT and RelA T505A lymphoma cells (Supplementary Figure S8C). Furthermore, in normal B cells no difference in *Clspn* expression was seen between WT and T505A or c-Rel^−/−^ cells (Supplementary Figure S8D), Taken together, this suggests that in Eμ-Myc lymphoma cells, mutation of RelA at T505 or loss of c-Rel results in a failure to induce *Clspn* mRNA levels in response to DNA replication stress.

Our analysis also revealed a high level of heterogeneity of *Clspn* mRNA expression in Eμ-Myc lymphoma cells, with many WT lymphomas showing similar levels of Claspin to those from Eμ-Myc/*Rela*^T505A^ or Eμ-Myc/*cRel*^−/−^ mice (Figure 8A). This suggested two distinct populations of WT Eμ-Myc lymphoma cells with high and low Claspin levels. Western blot analysis confirmed that Claspin protein levels in WT lymphomas corresponded to mRNA levels and were also reduced in the Eμ-Myc/*Rela*^T505A^ or Eμ-Myc/*cRel*^−/−^ lymphomas, with the exception of one T505A sample (Figure 8B). To see if the two populations of WT Eμ-Myc mice exhibited differential lymphoma progression we analysed the survival of Eμ-Myc mice with *Clspn* mRNA expression either below or above the median level (Figure 8A). This revealed that low levels of *Clspn* mRNA in WT mice were associated with significantly reduced survival (median onset 82 versus 138 days) (Figure 8C, Supplementary Figure S9A,B), suggesting that high levels of Claspin are protective against lymphomagenesis in this model. In contrast, analysis of the mRNA levels of other genes associated with the response to replication stress, *Atr*, *Atrip*, *Topbp1*, *Rad17* and *Chek1* showed no correlation with the survival times of Eμ-Myc mice (Supplementary Figure S8B). These data suggested that the low Clspn levels in Eμ-Myc/*Rela*^T505A^ or Eμ-Myc/*cRel*^−/−^ mice might, in part, account for the reduced survival times we see in these models (Figure 3A) [22].

### Lower Claspin levels may contribute to Eμ-Myc B-cell lymphoma CHK1i resistance

We next investigated whether lower Claspin levels might contribute to the resistance to CCT244747 treatment we observed in the reimplanted Eμ-Myc/*Rela*^T505A^ lymphomas. Here, we hypothesised that reduced Claspin levels would lead to less efficient activation of CHK1 by ATR, resulting in the reduced and altered phosphoproteomic signature we observed in response to CCT244747 treatment above (Figures 5 and 6). Western blot analysis confirmed reduced levels of Claspin protein in reimplanted Eμ-Myc/*Rela*^T505A^ lymphomas, while in Eμ-Myc/*cRel*^−/−^ extracts it was almost totally absent (Supplementary Figure S9C). In our parallel study, we have also observed reduced Claspin mRNA levels in isolates of the osteosarcoma cell line, U2OS that we have adapted over time to develop resistance to the CCT244747 [24].

**Figure 9.**
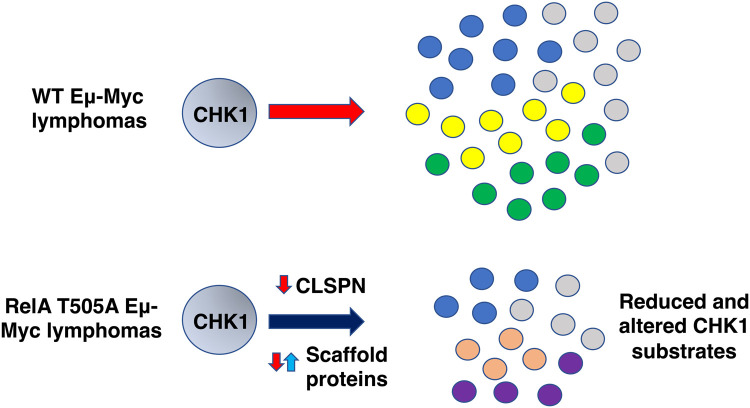
Model for reduced and altered CHK1 activity in RelA T505A Eμ-Myc lymphoma cells. Down-regulated CLSPN expression and changes to the levels of CHK1 interacting proteins combine to reduce the overall level of CHK1 activity and alter the range of substrates that can be phosphorylated.

To directly test whether reduced Claspin protein levels could confer resistance to CCT244747, we isolated primary ear fibroblasts from *Clspn*^+/−^ mice and WT littermates [25]. Western blot analysis confirmed that loss of a single allele of the *Clspn* gene resulted in reduced Claspin protein levels, without affecting the expression of ATR or CHK1 (Supplementary Figure S9D). Importantly, analysis of two independent isolates of fibroblasts confirmed partial resistance to CCT244747 treatment in *Clspn*^+/−^ cells (Figure 8D, Supplementary Figure S9E). This result was further confirmed by using siRNA to knock down Claspin expression in U2OS osteosarcoma cells (Supplementary Figure S9F), which elsewhere we have shown are sensitive to CCT244747 treatment but can be adapted to become CHK1i resistant [24]. Following siRNA knockdown of Claspin, cells were treated with CCT244747 and a clonogenic survival assay was performed. While knockdown of CLSPN itself reduced the number of surviving U2OS clones by ∼50%, these cells were now CCT244747 resistant (Figure 8E). Although other factors are likely to be involved both in the earlier onset of lymphoma and CCT244747 resistance we see in RelA T505A Eμ-Myc lymphomas (see below and [24,26]), these data suggest that a reduction in Claspin expression makes a significant contribution towards these phenotypes (Figure 9).

## Discussion

This manuscript confirms and significantly extends previous work from our group investigating the physiological function of the RelA T505 phosphosite. Initial studies demonstrated that phosphorylation of this site results in a pro-apoptotic form of RelA in response to induction of the ARF tumour suppressor or treatment with the chemotherapeutic drug cisplatin [15–17]. This contrasted with the more usual anti-apoptotic functions of RelA found following stimulation with inflammatory stimuli [45]. This early work implied that RelA T505 phosphorylation could lead to a tumour suppressing form of NF-κB in some contexts, which again contrasted with the more usual tumour-promoting activity ascribed to this pathway [4,6]. The identification of CHK1 as a putative RelA T505 kinase linked these atypical consequences of NF-κB activity to DNA replication stress, a common feature of cancer cells and to activation of oncogenes such as MYC in particular [21].

A limitation of these earlier studies was that they were restricted to the tools available at the time, such as overexpression of transiently transfected plasmids or reconstitution of *rela*^−/−^ MEFs, while being performed in a limited range of cell lines. Therefore, the significance of this pathway *in vivo* and to tumourigenesis itself remained unproven and hypothetical. To address this, we created the RelA T505A knockin mouse [18]. Our prediction was that a mutation of this site that prevented its phosphorylation would break a link between RelA, CHK1 and DNA replication stress, removing at least some tumour suppressing functions of NF-κB. Consequently, we should see earlier onset of tumourigenesis or reduced survival in cancer models where RelA plays a role. Indeed, in our first report using the RelA T505A mouse with the DEN model of hepatocellular carcinoma, this was what we observed [18]. Moreover, not only did we observe earlier onset of cancer in this study but tumours in RelA T505A mice were more malignant hepatocellular carcinomas compared with the adenomas predominantly seen in control mice at this time point [18]. However, despite observing higher levels of proliferation and indications of DNA damage in these RelA T505A tumours, we were unable to obtain mechanistic insights into the reasons why we found earlier onset of disease.

To learn more, about the consequences of the RelA T505A mutation on the DNA replication response in cancer, we decided to investigate its effect in a cancer model driven by the MYC oncogene, the Eμ-Myc model of B-cell lymphoma [19]. Since MYC is known to induce high levels of DNA replication stress (reviewed in [46]), we reasoned that this would provide an ideal system to obtain mechanistic insight into the importance of the RelA T505 phosphosite. However, it should be noted that since the T505 residue is within the RelA TAD, we cannot rule out other structural effects associated with mutating this site to alanine, as is the case with all phosphosite mutations. As predicted from our earlier work, we again saw reduced survival times in Eμ-Myc/*Rela*^T505A^ mice (Figure 3A). Due to the nature of this study, we cannot distinguish between earlier onset of lymphomagenesis versus development of a more aggressive lymphoma.

RNASeq and proteomic analysis revealed that the RelA T505A mutation has a major effect on the transcriptome and proteome of in Eμ-Myc/*Rela*^T505A^ lymphomas (Figures 4 and 5, Supplementary Figure S5 and Supplementary Data Files S1–S3). There was surprisingly little overlap between these datasets indicating a high level of post-transcriptional regulation (Supplementary Figure S5). Of the mRNAs whose expression was significantly changed in Eμ-Myc/*Rela*^T505A^ lymphomas, 19% (218/1139) were identified as potential direct RelA targets (Supplementary Figure S5A and Supplementary Data File S4). This list does include some known NF-κB targets and genes involved in the NF-κB pathway (e.g. Vcam1, Ikbkg, Tnfaip2, Irak1). Furthermore, of this gene list, 3 are also in the list of genes with known links to CHK1 (Poli, Rev1, Ddb2) we identified as having altered expression in Eμ-Myc/*Rela*^T505A^ lymphomas (Supplementary Figure S7D and Supplementary Data File S8). However, the potential importance of these and other potential target genes to the phenotype of the Eμ-Myc/*Rela*^T505A^ mice is unclear. It would be interesting in future studies to confirm the identity of direct RelA targets through ChIPSeq analysis. In addition, analysis of lymphoma cells at an earlier stage of development might reveal those target genes most likely to be defining the altered phenotype of the Eμ-Myc/*Rela*^T505A^ lymphomas.

The main focus of this report is how the RelA T505A mutation leads to a disruption of the DNA replication response in Eμ-Myc lymphomas and insights into this came from investigating the response to treatment with the CHK1i CCT244747 [29]. Tumours over expressing MYC have been previously identified as good targets for CHK1 inhibitors [29,39,47,48]. The high levels of DNA replication stress result in addiction to ATR/CHK1 signalling since this allows MYC driven tumour cells to survive high levels of genomic instability (reviewed in [49]). Moreover, we had previously shown that CCT245737 (SRA737), a CHK1i related to CCT244747 used here, effectively treated WT Eμ-Myc lymphomas [39]. Here we found that reimplanted Eμ-Myc/*Rela*^T505A^ lymphomas were resistant to CCT244747 treatment (Figure 3D–F). This implied either that the activity or function of CHK1 must be disrupted in the Eμ-Myc/*Rela*^T505A^ lymphomas or that they were no longer reliant on this pathway to survive.

Since development of resistance to kinase inhibitors is a major clinical problem [50], we decided to investigate the basis for this effect. To achieve this, RNA Seq and proteomic analyses after a single 8-h dose of CCT244747 was performed, so that the immediate response of these lymphoma cells to drug treatment could be determined. This revealed that the RelA T505A Eμ-Myc lymphoma cells response to CHK1 inhibition is different from WT cells (Figures 5–7). Most noticeably the number of total protein and phosphopeptide changes seen in RelA T505A cells are reduced. Moreover, further analysis revealed that the majority of these changes are different from those seen in WT cells. Therefore, while RelA T505A lymphoma cells do respond to CCT244747 treatment, they do so in a profoundly different way to wild-type cells. Further evidence that RelA T505A cells have fundamentally rewired their cell signalling pathways came from analysis of the intrinsic difference between Eμ-Myc WT and Eμ-Myc/*Rela*^T505A^ lymphoma cells without CCT244747 treatment. Here, it could be seen that there were a large number of differences in the total and phospho proteome (Figure 4D,E). Only 40 of these phosphosites overlapped with changes seen in WT Eμ-Myc cells upon CCT244747 treatment (not shown). This indicates that in contrast with our observations with Eμ-Myc/*cRel*^−/−^ lymphoma cells [24], RelA T505A cells only partially mimic WT cells that have been treated with CCT244747. Further analysis of some the intrinsic differences between RelA T505A and WT Eμ-Myc lymphoma cells have been performed elsewhere [24,26].

We cannot rule out that a component of the resistance we see to CCT244747 in Eμ-Myc/*Rela*^T505A^ lymphoma cells results from the up-regulation of ATP-binding cassette (ABC) transporters that have been shown to confer multidrug resistance in cancer (reviewed in [51]). Examination of our RNA Seq data reveals that of the 50 mRNA species encoding members of this family we detected, only three genes showed a significant (adj*P *< 0.05) change between the T505A and WT cells. These were ABCC4 (MRP4), which is up 0.47-fold (log_2_) in the T505A cells, ABCG2, which is up 0.84-fold (log_2_) in the T505A cells and ABCA7, which is down 0.49-fold (log_2_) in the T505A cells. In our proteomics data, of the ABC family members we could detect, none showed a statistically significant change in Eμ-Myc/*Rela*^T505A^ lymphomas. However, we think it is unlikely that changes in the expression of these genes are contributing to the effects we see. Firstly, as we show in this paper, the RelA T505A lymphomas do respond to CCT244747, albeit in a different manner to the WT cells. Secondly, in our parallel paper, where we investigate the compensatory bypass pathways that become activated in c-Rel null and Eμ-Myc/*Rela*^T505A^ lymphomas, we show that the RelA T505A lymphomas have increased sensitivity to drugs targeting the PI3K and PAK2 kinases [26].

To investigate whether expression of components of the ATR/CHK1 pathway itself were disrupted in RelA T505A cells, we performed qPCR analysis using RNA extracted from primary Eμ-Myc lymphomas. This experiment, originally performed before our RNA Seq and proteomic analysis of reimplanted Eμ-Myc lymphomas was completed, revealed lower levels of Claspin mRNA and protein expression in primary RelA T505A Eμ-Myc lymphomas, while ATR, CHK1, ATRIP, RAD17 and TOPBP1 were unaffected (Figure 8, Supplementary Figures S6 and S7). However, we observed that Claspin expression was variable in primary WT Eμ-Myc lymphomas, with both high and low expressing sub-populations (Figure 8A). Moreover, these populations behaved differently, with the lower expressing WT population displaying reduced survival times that closely matched both Eμ-Myc/*Rela*^T505A^ and Eμ-Myc/*cRel*^−/−^ mice (Figure 8, Supplementary Figures S8 and S9). Since we observed no defect in Claspin expression in normal B cells from RelA T505A and c-Rel^−/−^ mice (Supplementary Figure S8D), we propose that our data reveal a failure to induce Claspin under conditions of high DNA replication stress in the NF-κB mutant mice. And since Claspin is required for ATR phosphorylation and activation of CHK1 [52,53], this would lead to the altered checkpoint kinase signalling we observe. This defect, which also appears to be present in a subset of the WT Eμ-Myc lymphomas could then lead to earlier onset of lymphoma through increased genomic instability resulting in loss of tumour suppressor pathways [54]. Alternatively, loss of CHK1 activity results in the activation of bypass pathways in both Eμ-Myc/*Rela*^T505A^ and Eμ-Myc/*cRel*^−/−^ lymphomas, which we demonstrate elsewhere [26]. The effect of these could also contribute to earlier or more aggressive lymphoma development.

By western blot we also see reduced Claspin protein levels in reimplanted RelA T505A lymphoma extracts (Supplementary Figure S7C). Moreover, primary ear fibroblasts from the *Clspn* ^+/−^ mouse, together with U20S cells where Claspin expression has been reduced by RNA interference, displayed increased resistance to CCT244747 treatment (Figure 8D,E, Supplementary Figure S9E). Elsewhere we demonstrate that *Clspn* haploinsufficiency has significant physiological and developmental effects [25]. Therefore, a reduction in Claspin protein levels can account, at least in part, for the CCT244747 resistance we observe in reimplanted RelA T505A Eμ-Myc lymphomas (Figure 3D–F). However, from our RNA Seq and proteomic data we also see changes in other proteins either linked to or shown to bind directly to CHK1 (Figure 7D, Supplementary Data File S8). Although we did not directly examine these, it is likely that they also contribute towards altered CHK1 targeting and specificity in Eμ-Myc/*Rela*^T505A^ lymphomas.

Interrogation of our proteomic data largely supported these results, with levels of Claspin being significantly down-regulated in WT cells upon CHK1i treatment (*P* = 3.4 × 10^−4^) and in cRel^−/−^ compared with WT (*P* = 5.36 × 10^−4^) (Supplementary Data File S1). However, this was not seen in RelAT505A cells compared with WT (*P* = 0.85). Similarly, our RNA Seq analysis of these samples confirmed a reduction in *Clspn* mRNA in c-Rel^−/−^ lymphomas but again this was not the case with the RelA T505A Eμ-Myc lymphomas (Supplementary Data File S2). The reasons for this discrepancy are unclear. These could result from technical differences in protein extraction protocols, a contribution of wild-type cells in the reimplanted model while in the primary lymphomas all cells have the RelA T505A mutation, differences in growth or cell cycle characteristics between primary and reimplanted lymphomas or the reduced number of lymphoma samples used in the RNA Seq and proteomics analysis versus the primary lymphoma analysis. Regarding the latter possibility, we did not screen lymphomas used for RNA Seq and proteomics beforehand for Claspin expression. Given the variability we see in primary WT lymphoma cells (Figure 8A,B), we may, therefore, have inadvertently selected low Claspin expressing cells. In addition, we did select RelA T505A lymphomas that had appropriate lymph node homing characteristics (Supplementary Figure S3D) and this might have also influenced the data generated. Regardless, it is, therefore, likely that other factors can contribute to the CCT244747 resistance we see in RelA T505A lymphoma cells. For example, we also see reduced levels of the deubiquitinase USP1 in these cells [24] and up-regulation of the Pak1/Pak2 signalling pathway [26]. These changes may also contribute to the effects we see.

Despite these remaining uncertainties, this report reveals the major regulatory role that the RelA T505 phosphosite has as a regulator of the DNA replication stress response in an *in vivo* model of MYC driven lymphoma. Our current and previous data suggest a model in which phosphorylation of this site, under conditions of high DNA replication stress, is required to drive ATR/CHK1 pathway activity. This can be through inducing Claspin gene expression, thus ensuring efficient activation of CHK1 by ATR, as well as the other potential mechanisms discussed above. We propose two major consequences of this. In early stage cancer cells, T505 phosphorylated RelA will contribute towards DNA repair, thus preventing the genomic instability leading to further cancer-causing mutations. It will thus function as a tumour suppressor. However, when cancer cells escape this protective pathway and switch to becoming addicted to ATR/CHK1 activity, to allow them to cope with high levels of DNA replication stress, T505 phosphorylated RelA will contribute towards tumour survival. The consequences of mutating the RelA T505 site to alanine and preventing its phosphorylation are, therefore, two-fold. Increased genomic instability at the early stages of tumourigenesis, could lead to the earlier onset of cancer or development of a more aggressive phenotype (Figure 3A and [18]). In contrast, in late-stage cancer cells, this leads to CHK1i resistance and the activation of bypass pathways [26] that fundamentally rewire the cell signalling pathways of the cancer cell. By further understanding the molecular events in this model, we hope to develop biomarkers that could target the effective use of CHK1 inhibitors in the clinic and lead to the development of combinatorial or second line cancer therapies.

## Methods

### Ethics statement

All mouse experiments were approved by Newcastle University's Animal Welfare and Ethical Review Board. All procedures, including the of breeding genetically modified mice, were carried out under project and personal licences approved by the Secretary of State for the Home Office, under the United Kingdom's 1986 Animal (Scientific Procedures). Animals were bred in the Comparative Biology Centre, Newcastle University animal unit, according to the FELASA Guidelines.

### Mouse models

*RelA*^T505A^ knock in mice were generated by Taconic Artemis (Germany) using C57Bl/6 ES cells [18], C57Bl/6 mice used for reimplantation studies were purchased from Charles River. Male Eμ-Myc transgenic mice that were used as breeding stock were omitted from the survival analysis. In all experiments, the relevant pure C57Bl/6 (WT) strain was used as a control. No blinding of groups in mouse studies was performed. All mice were designated to an experimental group dependent on their genotype.

### Analysis of secondary lymphoid organ and blood cellular content

Spleens, inguinal lymph nodes, blood and peritoneal fluids were isolated from WT T505A mice. For blood samples, red blood cell lysis was performed using Pharm Lyse lysis buffer (BD) according to manufacturer's instructions. Following the generation of single-cell suspensions, total cell counts were calculated in the presence of trypan blue (Sigma–Aldrich) using a haemocytometer, followed by incubation with anti-CD16/CD32 Fc-Blocking antibody (clone 2.4G2 (BD Pharmingen)). Distinction between live and apoptotic/necrotic cells was performed based on staining with LIVE/DEAD Aqua (Life Technologies). For cell surface marker detection cells were stained with a combination of FITC, PE, APC, PerCP-Cy5.5, APC-Cy7 conjugated monoclonal antibodies (BD Pharmigen). For detection of Foxp3, intracellular staining was performed using a Fix/Perm kit (eBiosciences). See below for additional antibody information. All samples were acquired using a three laser FACS Canto II (BD Bioscience) and the data were subsequently analysed using FlowJo (version 9 or X; Treestar, U.S.A.).

**Antibodies used in flow cytometric analysis of lymphoid tissue cells**

**Table:** 

Target	Conjugate	(μg/ml)	Source
Mouse CD19	FITC	1	BD Pharmingen
Mouse F480	PE	0.5	Biolegend
Mouse CD11b	APC-Cy7	1	BD Pharmingen
Mouse CD3	APC	1	BD Pharmingen
Mouse CD4	FITC	1	BD Pharmingen
Mouse CD8	FITC	1	BD Pharmingen
Mouse CD5	PerCP-Cy5.5	1	BD Pharmingen
Mouse CD138	PE	1	BD Pharmingen
Mouse CD25	PE	1	BD Pharmingen
Mouse FoxP3	FITC	2	BD Pharmigen
Mouse CD11c	APC	2	BD Pharmingen

### Drugs and compounds

CCT244747 was synthesised as described [29]. MK8776 was purchased from (S2735 Selleckchem, U.K.). Etoposide was purchased from (E1383, Scientific Lab Supplies Nottingham, U.K.), SN38 (2684) and Mitomycin C (3258) were obtained from (R&D systems, Abingdon, U.K.). All other compounds were purchased from Sigma–Aldrich.

### Generation of mouse embryo fibroblasts (MEFs)

Heterozygote *RelA*^T505A^ mice were bred to produce a mixture of WT and *RelA*^T505A^ homozygote littermates. MEFs were isolated as follows. Internal torso connective tissue from 13.5-day embryos was washed in sterile PBS and minced in 1× Trypsin (Invitrogen) for 15 min at 37°C. Following repeated pipetting to break up large tissue fragments, the cell pellet was re-suspended in DMEM (BE12-604Q, Lonza) supplemented with 20% Fetal Bovine Serum (10270106, FBS) (Gibco, Paisley, U.K.) and 50 U/ml penicillin/streptomycin (PS) (09-757F, Lonza), and incubated at 37°C in a 5% CO_2_ humidified atmosphere. Once cells reached 90% confluency, they were sub-cultured in 75 cm^2^ flasks and considered as passage 1. Cells were then cultured following the standard 3T3 protocol [56]. Cells were considered immortalised beyond passage 14, but not used in experiments beyond passage 25.

### Generation of Ear fibroblasts

Ear Fibroblasts were generated as previously described [57]. Briefly, ear punch biopsies (two per mouse) were transported and stored in DMEM containing 10% FBS on ice. Punches were washed three times with serum-free DMEM, finely cut and incubated for 2 h at 37 °C in 2 mg/ml collagenase A (COLLA-RO, Sigma–Aldrich) in DMEM. A single-cell suspension was obtained by repeated pipetting and passing through a 25G needle. Cells were centrifuged for 10 min at 95 ***g*** and cultured in Advanced DMEM/F-12 (12634010, Invitrogen) plus 10% FBS, 50 U/ml PS (Lonza), 2 mM l-glutamine (BE17-605E, Lonza) at 37°C in a 5% CO_2_ humidified atmosphere. Each isolate was derived from a separate mouse.

### Apoptosis assays

*RelA*^T505A^ or WT MEFs were grown in 96-well plates and treated for 24 h with the appropriate concentration of Cisplatin (P4394) (4 μg/ml), Etoposide (15 μM), Hydroxyurea (H8627) (0.5 mM), SN38 (5 μM) or Mitomycin C (1 μg/ml). Cells were then harvested, lysed and assayed for Caspase-3 activity using the CaspACE Assay System (G8090, Promega, Southampton, U.K.), according to manufacturer's guidelines. Samples were normalised to their protein concentration using the Pierce BCA Protein Assay Kit (23225, ThermoFisher Scientific, U.K.).

### Cell viability assays

Immortalised WT or *RelA*^T505A^ MEFs (5 × 10^3^) or primary WT or *Clspn*^+/−^ ear fibroblasts (2.5 × 10^4^) per well were seeded into 96-well plates. Increasing concentrations of the novel CHK1i, CCT244747 (ICR, Sutton, U.K.), or MK8776 or solvent controls were added to three replicate wells. After 96 h, viability was quantified using the PrestoBlue Cell Viability Reagent (A13261, ThermoFisher Scientific, U.K.), according to manufacturer's instructions.

### Cell survival assays

Exponentially growing immortalised WT or *RelA*^T505A^ MEFs were treated for 24 h with 1 μM or 5 μM CHK1i, CCT244747 or MK8776 or solvent controls before re-seeding onto Petri dishes at known cell number (1000, 2500 or 5000 cells/dish). Colonies were fixed 14 days later with methanol : acetic acid (3 : 1) and stained with 0.4% (w/v) Crystal Violet. Cloning efficiencies were normalised to untreated controls.

### siRNA knockdown transfections and survival assays

U2OS cells were transfected with 5 nM siRNA targeting Claspin (L-005288-00) (ON-TARGET plus Smart pool, Dharmacon) or a Non-specific siRNA control (D-001810-00) using Interferin PolyPlus transfection reagent (409-50, VWR), according to manufacturer's protocols. Cells were used in cell survival assays 48 h post-transfection, once target depletion had been confirmed. Forty-eight hours post-transfection, cells were treated for 24 h with 1 μM CHK1i, CCT244747, or solvent controls before re-seeding onto 6-well plates at known cell number (750, 1500 or 2500 cells/well). Colonies were fixed 21 days later with methanol : acetic acid (3 : 1) and stained with 0.4% (w/v) Crystal Violet. Cloning efficiencies were normalised to solvent treated non-specific siRNA controls.

### Gene expression analysis using quantitative real-time PCR

Total RNA was puriﬁed from Eμ-Myc, Eμ-Myc*/RelA^T505A^* or Eμ-Myc/*c-Rel*^−/−^ tumour cell pellets using the PeqGold total RNA extraction kit (12-6834-02, Peqlab). Where snap frozen tumours tissue was used, total RNA was extracted by homogenisation using Precellys 24 ceramic mix bead tubes (431-0170, Stretton Scientific Ltd) in a Precellys 24 benchtop homogeniser (Stretton Scientific Ltd) at 6500 rpm for 30 s. Following this, samples were passed through Qiashredders (79656, Qiagen, Crawley, U.K.) and RNA was purified using the Qiagen RNeasy mini kit (74004) according to manufacturer's instructions.

RNA was measured for purity and concentration with the NanoDrop1000 (ThermoFisher Scientific) and reverse transcribed using the Quantitect Reverse transcription Kit (Qiagen) according to manufacturer's instructions. Quantitative real-time PCR was performed on 20 ng cDNA, in triplicate, using predesigned Quanititect Primer assays (Qiagen) to the following murine genes; *Clspn* (QT00154609), *Atr* (QT00284172), *Atrip* (QT00131915), *Rad17* (QT00494823), *Topbp1* (QT00155008), *Chek1* (QT00109179)*.* These samples were run and analysed on a Rotor-gene Q system (Qiagen), using murine *Gapdh* (QT01658692) or *Rpl13a* primers (QT00267197) as an internal control. All CT values were normalised to *Gapdh* or *Rpl13a* levels as indicated in the figures.

### Western blotting

Whole cell extracts were prepared from WT or *RelA*^T505A^ MEFs, or Eμ-Myc, Eμ-Myc/*c-Rel*^−/−^ or Eμ-Myc*/RelA^T505A^* extracted direct from snap frozen pieces of tumour. In the case of snap frozen tumours, these were lysed in PhosphoSafe™ Extraction Reagent (71296, Merck Millipore) using the Precellys24 ceramic mix bead tubes (Stretton Scientific Ltd) in a Precellys®24 homogeniser (Stretton Scientific Ltd) at 6500 rpm for 30 s, then extracted according to the PhosphoSafe™ Extraction Reagent manufacturer's instructions. In the case of tumour cell suspensions or MEFs, cell pellets were washed with ice-cold PBS, and lysed using PhosphoSafe™ Extraction Reagent (Merck-Millipore, Watford, U.K.), according to manufacturer's protocols. Ear fibroblasts were washed with 1× PBS and lysed with 300 μl Urea lysis buffer (8 M Urea, 50 mM Tris [pH 8.0], 300 mM NaCl, 50 mM Na_2_HPO_4_, 0.5% NP-40, 1 mM PMSF, supplemented with protease inhibitors) per 10 cm plate. Lysates were incubated on ice for 15 min before being clarified by centrifugation 13 000 rpm at 4°C for 10 min. Protein quantification was undertaken using the BCA protein assay, and samples resolved by standard denaturing SDS–PAGE gels. Samples were transferred onto PVDF membrane (GVWP04700, Merck-Millipore) before being probed with the primary antibody. Horseradish peroxidase-conjugated secondary antibodies and enhanced chemiluminscence reagent (Thermo-scientific, U.K.) were used for detection.

### Antibodies

Antibodies used were c-Rel (sc-71 Santa Cruz), c-Myc (sc-42 Santa Cruz), ATR (sc1887 Santa Cruz), RelA (sc-372 Santa Cruz), RelB (4954 Cell Signaling), p105/p50 (ab7971 Abcam), p100/p52 (4882 Cell Signaling), β-Actin (A5441 Sigma), CHK1 (phospho S345) (2341 Cell Signaling), CHK1 (2360 Cell Signaling) RelA (phospho 536) (3031 Cell Signaling), active caspase 3 (9664 Cell Signaling), γH2AX (9718 Cell Signaling), RPA2/RPA32 Ser 33 (10148 Cell Signaling), RPA2/RPA32 (52488 Cell Signaling), ATRIP (11327-1-AP Proteintech), Rad17 (13358-1-AP Proteintech), ATR (13934 Cell Signaling), Karyopherin Beta (Santa Cruz sc-137016). Antibodies to the murine form of Claspin was generated by Moravian Biotechnologies. Anti-rabbit IgG (A6154 Sigma and 7074 Cell Signaling) and anti-mouse IgG (A9044 Sigma) HRP-linked secondary antibodies were used for western blot detection.

### Eμ-Myc mice studies

Eμ-Myc*/RelA*^T505A+/−^ offspring were generated by mating *T505A* female mice with Eμ-Myc male mice, further Eμ-Myc/*RelA*^T505A^ mice were generated by crossing Eμ-Myc*/T505A*^+/−^ males with *T505A* female mice. Only group housed Eμ-Myc/*RelA*^T505A^ males were included as the cohort for this analysis to minimise any potential effects from environmental and endogenous oestrogens. To perform survival analysis, Eμ-Myc transgenic mice were monitored daily and were sacrificed at pre-determined endpoints, defined as the animal becoming moribund, losing bodyweight/condition and/or having palpable tumour burden at any lymphoid organ site.

Survival analysis (Kaplan Meier analysis) was carried out using GraphPad Prism (Version 5.0) and signiﬁcance determined using the log-rank (Mantel–Cox) test. Moribund mice were necropsied and single-cell suspensions were prepared from tumour-bearing organs. Mice were humanely sacrificed by cervical dislocation. No anaesthesia was used at any point during any studies described. Briefly, lymph nodes, spleen or thymus were homogenised through a cell strainer, and single-cell suspension collected in DMEM (Lonza) supplemented with 10% FBS, 5 mM l-glutamine, 5 mM sodium pyruvate, 1μM l-asparagine and 50 μM β-mercaptoethanol (Sigma–Aldrich). These cell suspensions were then used for downstream analyses or frozen in 90% FBS/10% DMSO for long term storage and transplantation.

Technical note: This study does not include analysis of survival in Eμ-Myc/*Clspn*^+/−^ mice. We were advised that the Director of the Newcastle University Faculty of Medical Sciences Comparative Biology Centre had serious ethical and 3Rs concerns with this option, due to the number of mice required to generate a sufficiently powered cohort of male Eμ-Myc/*Clspn*^+/−^ mice. Female *Clspn*^+/−^ mice have reduced litter sizes (2 versus 5 for wild-type mice in 100 days), lengthened time to first litter (44.5 days versus 22 days for wild-type mice) and reduced average pups per litter (3.8 versus 7.2 for wild-types) [25]. Eμ-Myc mice can only be bred as heterozygote transgenes (homozygous are non-viable) and Eμ-Myc females cannot be used to carry litters due to the early onset of disease. Consequently only 1 in 16 of the *Cslpn*^+/−^ mice (female) ×  Het Eu-Myc mice (male) would be male and have the Eμ-Myc/*Clspn*^+/−^ genotype. We would, therefore, only obtain a suitable male mouse approximately once every four successful matings.

### Reimplantation studies

For tumour therapy studies, 2 × 10^6^ Eμ-Myc or Eμ-Myc/*RelA*^T505A^ tumour cells from male mice were transplanted intravenously via the lateral tail vein into 8-week-old male C57BL/6 recipients. Mice were monitored daily using parameters such as their bodyweight and food and water consumption to assess disease progression. Mice were necropsied when they became moribund and the tumour burden assessed.

Oral administration of the CHK1i, CCT244747 (ICR, Sutton, U.K.), or vehicle control (65% PEG-400, 20% Tween-20, 10% H_2_O, 5% DMSO (all Sigma–Aldrich)) was initiated when tumours became palpable (approximately 10 days after inoculation of Eμ-Myc or Eμ-Myc/*RelA*^T505A^ cells. CCT244747 was given as a single agent, bolus dose (100 mg/kg p.o.) for 9 consecutive days. Lymphoid tumour burden and final tumour weights were measured at necropsy 24 h after the final dose.

### Proteomics and analysis

Tissue extracts were prepared from snap frozen pieces of Eμ-Myc, or Eμ-Myc/*RelA*^T505A^ splenic tumours. Briefly, tissue samples were suspended in 100 mM triethylammoniumbicarbonate (TEAB) with a mixture of protease and phosphatase inhibitors (cOmplete Mini EDTA-free protease inhibitor cocktail plus PhoSTOP phosphatase inhibitor cocktail, both obtained from Roche), homogenised by bead beater, and sonicated on ice. Lysed extracts were incubated with 0.1% (w/v) Rapigest SF (Waters) for 10 min at 80°C, left to cool, and incubated for 10 min on ice with Benzonase endonuclease (Merck Millipore) to digest nucleic acids. Samples were centrifuged (14 000 ***g***, 10 min at 4°C) to pellet cell debris. Protein concentration of the clarified lysate was ascertained by Bradford assay. Protein (200 µg) from each sample was aliquoted for protein digestion.

Disulfide bonds were reduced (4 mM DTT in 100 mM TEAB, 10 min at 60°C) and free cysteines alkylated with iodoacetamide (14 mM in 100 mM TEAB, for 30 min, RT in the dark). Iodoacetamide was quenched by addition of DTT to a final concentration of 7 mM. Proteins were digested with 2% (w/w) trypsin overnight at 37°C with gentle agitation. Resultant peptides were labelled with TMT 6-plex reagents (Thermo Scientific) at an 8 : 1 tag : protein ratio as per the manufacturer's instructions, with labels assigned to samples randomly for the first biological replicate and shifted for each subsequent replicate. The labelling reaction was quenched by addition of 0.3% (v/v) hydroxylamine (Thermo Scientific) in 100 mM TEAB. TMT labelled peptides were mixed and dried to completion by vacuum centrifugation before re-suspending in 100 mM TEAB/ 1% TFA to hydrolyse the Rapigest SF (RT, 10 min). Insoluble Rapigest SF cleavage product was removed by centrifugation (13 000 *g* for 15 min at 4°C), and the sample desalted using C18 spin columns (Pierce, #89852) as per the manufacturers protocol, prior to strong cation exchange using stage tips (packed in-house with five disks per 200 µl tip as described previously [58] (Empore Supelco 47 mm Cation Exchange disk). Each mixed labelled peptide sample was split across eight tips, with peptides passed through the equilibrated stage tips twice. Bound peptides were eluted with 5% NH_4_OH (3 × 100 µl) and dried to completion using a vacuum centrifuge.

Peptides were fractionated using basic reverse-phase liquid chromatography as described [58], with 65 fractions collected, partially dried by vacuum centrifugation, and concatenated into five pools. For each pool, 5% was aliquoted and dried to completion prior to MS analysis. The remaining 95% was subjected to TiO_2_-based phosphopeptide enrichment, as described previously [43].

Total protein and phosphopeptide-enriched fractions were analysed by LC–MS/MS using an UltiMate 3000 RSLCTM nano system (Dionex) coupled in-line with a Thermo Orbitrap Fusion Tribrid mass spectrometer (Thermo Scientific). Peptides were loaded onto the trapping column (PepMap100, C18, 300 µm × 5 mm, Thermo Scientific) using partial loop injection with 2% acetonitrile (ACN), 0.1% TFA at a flow rate of 9 µl/min for 7 min. Peptides were resolved on an analytical column (Easy-Spray C18, 75 µm × 500 mm, 2 µm bead diameter) using a gradient from 96.2% A (0.1% formic acid):3.8% B (80% ACN, 0.1% formic acid) to 50% B over either 120 min (single injection for phosphopeptide-enriched samples and two injections for total protein samples) or 240 min (single injection for total protein samples only) at a flow rate of 300 nl/min. Full MS1 spectra were acquired in the Orbitrap over *m/z* 375–2000 (60 K resolution at *m*/*z* 200), with a maximum injection time of 50 ms and an ACG target of 4e5 ions. Data-dependent MS2 analysis was performed using a top speed approach (3 s cycle time) with peptides fragmented by collision-induced dissociation [59] at a normalised collision energy [7] of 35%, with fragment ions detected in the ion trap (maximum injection time of 50 ms, ACG target of 1e4). Following acquisition of each MS2 spectrum, a synchronous precursor selection (SPS) MS3 scan was performed on the top 10 most intense fragment ions, with SPS-MS3 precursors fragmented using higher energy collision-induced dissociation (HCD), at an NCE of 65%, and analysed using the Orbitrap over *m/z* 100–500 (50 K resolution at *m*/*z* 200) with a maximum injection time of 105 ms and an ACG target of 1e5 [60,61].

Analysis of MS data, with quantification of TMT reporter ion distributions, was performed using Proteome Discoverer 2.4 (PD 2.4) in conjunction with MASCOT (v2.6) and Percolator. For peptide identification from MS2 spectra, raw data files were converted to mzML format and searched in MASCOT against the Mouse UniProt reviewed database (Downloaded 25/04/2018; 16 966 sequences) with parameters set as follows: MS1 tolerance of 10 ppm; MS2 tolerance of 0.6 Da; enzyme specificity was set as trypsin with two missed cleavages allowed; carbamidomethylation of cysteine and TMT 6-plex modifications (on peptide N-termini and lysine side chains) were set as fixed modifications; oxidation of methionine and acetylation of protein N-termini were set as variable modifications, with the addition of phosphorylation (at serine, threonine or tyrosine residues) for phosphopeptide-enriched samples. Percolator was used for control of FDRs with a target FDR of 0.05. For phosphopeptide-enriched samples, the ptmRS node, operated in phosphoRS mode, was added to the PD 2.4 workflow for phosphosite localisation. In parallel with peptide identification, relative quantification of TMT 6-plex reporter ions was performed in PD 2.4 using the ‘Reporter ions quantifier’ node, to quantify reporter ions from MS3 spectra with a peak integration tolerance of 20 ppm using the ‘most confident centroid’ integration method.

Quantitative ratios were calculated for each biological replicate, log_2_ transformed and, for all proteins/phosphopeptides quantified in at least 3 out of 5 bioreps, statistical analysis was performed in R using the LIMMA package, with a *P *≤ 0.05 significance cut off.

Please note that data from control samples from the long term and acute CCT244747 WT Eμ-Myc mice is also used in the analysis of changes in c-Rel^−/−^ Eμ-Myc lymphomas described elsewhere [24]. Consequently, this description of the methods is duplicated in that paper. Moreover, some figures using these control samples are also duplicated in that study. These are clearly indicated in figure legends. We have compiled supplementary data from proteomics analysis in the study into a single file (Supplementary Data File S1), which is also attached to the other papers that analyse this data [24,26].

### RNA-Seq and analysis

RNA was extracted as described above and sample quality analysed using Tapestation automated electrophoresis (Aglient) according to manufacturer's instructions. Sample RNA integrity number (RIN) score exceeded six in all cases. mRNA-Seq libraries were prepared using the Illumina TruSeq Stranded mRNA kit following manufacturer's reference guide and sequenced on an Illumina NextSeq 500 high-output 75 cycle flow cell, generating 25 million 75 bp single reads per sample. The raw sequence data quality was first inspected using FastQC and MultiQC [62] using Release M20 (GRCm38.p6) of the mouse genome (for the mouse samples) and Release 31 (GRCh38.p12) of the human genome (for the human samples).

The quantification files were imported into R for gene-level analyses using tximport [63] and the differential gene expression analyses were carried out using DESeq2 [64]. The data have been deposited on ENA (https://www.ebi.ac.uk/ena/submit/sra/#home) with the accession number PRJEB45284.

Please note that data from control samples from both the long term and acute CCT244747 treated WT Eμ-Myc mice is also used in the analysis of changes in c-Rel^−/−^ Eμ-Myc lymphomas described elsewhere [24]. Data from the T505A Eμ-Myc mice is also analysed in our analysis of bypass pathways [26]. Consequently, this description of the methods is duplicated in that paper. Moreover, some figures using these control samples are also duplicated in that study. These are clearly indicated in figure legends. We have compiled supplementary data from RNA Seq analysis in the study into single files (Supplementary Data File S2 and S3), which are also attached to the other papers that analyse this data [24,26].

### STRING, Venn diagram and gprofiler analysis

STRING analysis was performed using version 11.0 at https://string-db.org/ [65]. Where indicated CHEK1 was manually added to the protein list to determine connections to phosphoproteins identified from the proteomics analysis. Details on proteins analysed and connections are in Supplementary Data File S7. Venn diagram analysis was performed using https://www.biovenn.nl/index.php. To identify putative RelA binding sites from genes with altered expression in the RNA Seq analysis, analysis was performed at the Gprofiler website (https://biit.cs.ut.ee/gprofiler/gost) using the TRANSFAC database search setting. Gene symbols were pasted into Grpolfiler and searched as mus musculus organism. User threshold set to *P*-value < 0.05.

### Statistical analysis

GraphPad Prism software (http://www.graphpad.com, V6.0) was used for statistical analysis. Except where stated in figure legends, unpaired *t*-tests or One-way ANOVA were used to calculate *P* values (*P*-values of *P* < 0.05 were considered signiﬁcant).

## Data Availability

The mass spectrometry proteomics data have been deposited to the ProteomeXchange Consortium (http://proteomecentral.proteomexchange.org) via the PRIDE partner repository [55] with the dataset identifiers Project accession: PXD026203 and Project DOI: 10.6019/PXD026203. RNASeq data have been deposited on ENA (https://www.ebi.ac.uk/ena/submit/sra/#home) with the accession number PRJEB45284. The authors are happy to provide all original data, and for this to be shared on Figshare as appropriate.

## Open Access

Open access for this article was enabled by the participation of University of Liverpool in an all-inclusive *Read & Publish* agreement with Portland Press and the Biochemical Society under a transformative agreement with JISC.

## Abbreviations

ABC: ATP-binding cassette
ACN: acetonitrile
CHK1: Checkpoint kinase 1
CHK1i: CHK1 inhibitor
DEN: N-nitrosodiethylamine
FDR: false discovery rate
MEFs: mouse embryonic fibroblasts
PTMs: post-translational modifications
RPA: replication protein A
SPS: synchronous precursor selection
TEAB: triethylammoniumbicarbonate
TMT: tandem mass tag
WT: wild-type
