# Supplementary material for: Mutation of the RelA(p65) Thr505 phosphosite disrupts the DNA replication stress response leading to CHK1 inhibitor resistance
Source: Biochem J. 2022 Oct 14;479(19):2087–113. doi: 10.1042/BCJ20220089 (PMC9704643; doi:10.1042/BCJ20220089)

Supplementary Information

Mutation of the RelA(p65) Thr505 phosphosite disrupts the DNA replication stress response leading to CHK1 inhibitor resistance

Jill E. Hunter[1#], Amy E. Campbell[2#], Jacqueline A. Butterworth, Helene Sellier[1], Nicola L. Hannaway[1], Saimir Luli[3], Achilleas Floudas[3], Niall S. Kenneth[4], Adam J. Moore[1], Philip J. Brownridge[2], Huw D. Thomas[3], Jonathan Coxhead[1], Leigh Taylor[1] Peter Leary[4], Megan S.R. Hasoon[4] Andrew M. Knight[3], Michelle D. Garrett[6], Ian Collins[7], Claire E. Eyers[2*] and Neil D. Perkins[1*]

[1] Newcastle University Biosciences Institute
Wolfson Childhood Cancer Research Centre
Level 6, Herschel Building
Newcastle University
Brewery Lane
Newcastle upon Tyne, NE1 7RU, UK
[2]Centre for Proteome Research, Department of Biochemistry and Systems Biology,
Institute of Systems, Molecular and Integrative Biology,
University of Liverpool,
Liverpool L69 7ZB, U.K.
[3]Newcastle University Clinical and Translational Research Institute
Preclinical In Vivo Imaging
Faculty of Medical Sciences
Newcastle University
Newcastle Upon Tyne, NE2 4HH, UK
[4]Department of Molecular Physiology and Cell Signalling.
Institute of Systems, Molecular and Integrative Biology,
University of Liverpool,
L69 7ZB.
[5]Bioinformatics Support Unit,
Faculty of Medical Sciences
Newcastle University
Newcastle Upon Tyne, NE2 4HH, UK
[6]School of Biosciences,
Stacey Building,
University of Kent,
Canterbury, Kent, CT2 7NJ, UK
[7]The Institute of Cancer Research
Sutton, SM2 5NG, UK

[#]These authors contributed equally to this work
* corresponding authors

Tel. 0191 2082245 Email: neil.perkins@ncl.ac.uk

Tel. 0151 794424 mailto:Claire.Eyers@liverpool.ac.uk

Supplementary figure legends

Table S1: Mean cell counts from spleen and inguinal lymph nodes of WT and RelA T505A mice. Data analysed using a two-tailed Students t-test.

Table S2: Flow cytometric analysis of spleen, blood and inguinal lymph node cell populations of 6 x WT and 6 xT505A 12-week old male littermates. Peritoneal fluid control data is not shown. Data analysed using a two-tailed Students t-test.

Figure S1:

(A) *RelA*^{T505A} MEFs are resistant to apoptosis resulting from treatment with inducers of DNA replication stress. CASPASE 3 activity assay in immortalised WT and *RelA*^{T505A} MEFs after treatment with Cisplatin (4 μg/ml), or Etoposide (15 μM). Drugs were added for 16 hours and the results shown are the mean + SEM from 3 separate repeat experiments.

(B) *RelA*^{T505A} MEFs are resistant to Cisplatin induced apoptosis. Western blot analysis of full length and cleaved CASPASE 3 in immortalised wild type (WT) and RelA T505A MEFs after treatment with the DNA damaging agent, Cisplatin (4 μg/ml).

(C) Western blot analysis of the NF-κB subunits, c-REL, RELA, RELB, p100/p52, p50 together with c-MYC in extracts prepared from WT and *RelA*^{T505A} MEFs.

(D) The actin cytoskeleton is altered in *RelA*^{T505A} MEFs. T505A mutation increases filamentous actin fibres. Cells were stained with Alexa Flour 488 phalloidin and DAPI, and F-actin was examined using microscopy.

Figure S2:

(A) *RelA*^{T505A} MEFs are resistant to CHK1 inhibitor treatment. Cell viability (Prestoblue assay) in WT and *RelA*^{T505A} MEFs following treatment with increasing concentrations of the CHK1 inhibitor, CCT244747 for 72 hours.

(B) Increased clonogenic survival in *RelA*^{T505A} MEFs following Chk1 inhibitor treatment Clonogenic survival in WT and *RelA*^{T505A} MEFs following either treatment with either 1 μM (p=0.0032 ** Unpaired Student's T-test) or 5 μM (p=0.0504 Unpaired Student's T-test) of the Chk1 inhibitor, MK8776.

Figure S3:

(A & B) IHC analysis (A) and representative images (B) (10X magnification) of Eμ-Myc and Eμ-Myc/*Rela*^{T505A} lymph nodes stained with an antibody against γH2AX and counterstained with heamatoxylin. Brown staining indicates cells positive for γH2AX. Quantification of γH2AX positive pixels by IHC analysis (A) in Eμ-Myc and Eμ-Myc/*Rela*^{T505A} lymph nodes. Each dot represents one mouse and at least blinded 5 fields of view were analysed per mouse (p=0.0114 * Unpaired Student's t-test). The red dot in each case illustrates the mouse shown in (A).

(C) Western blot analysis of the NF-κB subunits, c-REL, RELA, RELB, p100/p52, p50 together with c-MYC in extracts prepared from Eμ-Myc and Eμ-Myc*/Rela*^{T505A} mouse tumorigenic spleens.

(D) Pie charts showing the homing of E*μ*-Myc (n=12) and Eμ-Myc*/Rela*^{T505A} (n=8) lymphoma cells following re-implantation. H&E images (10x objective) are also shown from the liver of WT and T505A re-implants. High levels of purple staining in the livers of mice reimplanted with T505A lymphomas suggest an infiltration of tumour B-cells.

Figure S4:

(A) Schematic showing how spontaneous tumours from Eμ-Myc mice are harvested, stored, re-implanted and used for efficacy studies

(B) Line graphs showing the mean response of the five reimplanted Eμ-Myc and four Eμ-Myc*/Rela*^{T505A} (orange) tumours and their response to CCT244747 in further lymphoid organs. Each of the tumours was implanted into 6 syngeneic recipient C57Bl/6 mice, 3 were treated

with CCT244747 (100 mg/kg p.o), and 3 with vehicle control, for 9 days once tumours became palpable. A response was defined as a significant reduction (or increase) in tumour burden ($P<0.05$) using unpaired Student's t-tests. Please note that data from WT Eμ-Myc lymphomas used here are replicated in another manuscript [24], where they are used for comparison with data from Eμ-Myc*/cRel-/-* lymphomas.

(C) Schematic illustrating the workflow for proteomics experiments. Splenic tumours from Eμ-Myc or Eμ-Myc*/Rela*^{T505A} mice were necropsied 8 hours post-treatment with either CHK1 inhibitor CCT244747 or vehicle control. Proteins were extracted and digested with trypsin prior to peptide labelling with tandem mass tags (TMT). Differentially labelled peptides from each treatment condition were mixed then fractionated via basic reverse-phase liquid chromatography, initially into 65 fractions which were concatenated into 5 pools. For each pool, 5% of the material was analysed by LC-MS/MS to obtain relative-quantification of total protein levels whilst the remaining 95% was subject to titanium dioxide (TiO_2)-based phosphopeptide enrichment prior to LC-MS/MS analysis for phosphoproteomic analysis.

Figure S5

(A) Volcano plot of putative RelA target genes. Potential RelA target genes were identified from the TRANSFAC database using gprofiler (https://biit.cs.ut.ee/gprofiler/gost) (Supp Data File 4). The volcano plot depicts the log2 fold change in the expression of these genes in Eμ-Myc*/Rela*^{T505A} lymphomas versus wild type Eμ-Myc lymphomas using data from RNA Seq analysis (Supp Data File 2).

(B) Venn diagram showing the overlap between proteins with phosphorylation changes and the changes observed in the total proteome in Eμ-Myc*/Rela*^{T505A} versus wild type Eμ-Myc lymphomas (see also Supp Data File 6).

(C) Venn diagram showing the overlap between mRNA changes and the changes in the total proteome in Eμ-Myc*/Rela*^{T505A} versus wild type Eμ-Myc lymphomas (see also Supp Data File 6).

Figure S6

Wider STRING analysis showing that a cluster of proteins associated with CHK1 had down-regulated phosphorylation was upon CCT244747 treatment in Eμ-Myc WT tumours after a single dose of the CHK1i. Boxed area indicates region shown in Fig 2B. See also Supp Data File 7.

Figure S7:

(A) Table detailing the 26 genes which showed differential expression in the Eμ-Myc WT tumours following acute CCT244747 treatment by RNA-Seq analysis. See also Supp data files 2 & 3.

(B) Venn diagram depicting the overlap between phosphoproteins that display a change in phosphorylation in Eμ-Myc/*Rela*^{T505A} lymphomas in response to CCT244747 not seen in WT Eμ-Myc lymphomas and the difference in total proteome between Eμ-Myc/*Rela*^{T505A} versus wild type Eμ-Myc lymphomas (no CCT244747 treatment). See also Supp Data File 6.

(C) Venn diagram depicting the overlap between phosphoproteins that display a change in phosphorylation in WT Eμ-Myc lymphomas in response to CCT244747 not seen in Eμ-Myc/*Rela*^{T505A} lymphomas and the difference in total proteome between Eμ-Myc/*Rela*^{T505A} versus wild type Eμ-Myc lymphomas (no CCT244747 treatment). See also Supp Data File 6.

(D) Table showing genes/proteins whose expression is different between Eμ-Myc/*Rela*^{T505A} versus wild type Eμ-Myc lymphomas (no CCT244747 treatment) and where String analysis (https://string-db.org/) links them to CHK1. Also shown are those proteins on this list where Biogrid (https://thebiogrid.org/) identifies them, or a related protein, as directly interacting with CHK1. See also Supp Data File 8.

Figure S8

(A) Schematic diagram illustrating the activation of CHK1 by ATR following DNA damage or replication stress, and the role of Claspin as an essential adaptor protein.

(B) Other components of the ATR/CHK1 signalling pathway are not affected in Eμ-Myc/*Rela*^{T505A} mice and do not correlate with onset of lymphoma. Q-PCR data (C) showing relative *Atr*, *Rad17*, *Atrip* , *Topbp1* and *Chek1* expression in tumorigenic spleens from Eμ-Myc (for c-Rel analysis n=20 A-D, n=11 E, for *Rela*^{T505A} analysis n=13 A-D, n=6 E), Eμ-Myc/*c-Rel-/-* (n=11) and Eμ-Myc/*Rela*^{T505A} (n=8) mice. Kaplan Meier survival analysis (D) of Eμ-Myc mice with below and above the median levels of *Atr, Rad17, Atrip, Topbp1* and *Chek1* mRNA (n=20 mice), and *Topbp1* (n=11 mice). No correlation with overall survival is seen.

(C) Western blot showing the expression of RAD17, ATRIP and ATR in extracts from reimplanted Eµ-Myc*/Rela*^{T505A} and wild type Eµ-Myc lymphomas. ACTIN or KARYOPHERIN B are used as loading control as appropriate.

(D) *Clspn* mRNA levels are not affected in non-tumorigenic B-cells from *Rela*^{T505A} mice. Q-PCR data showing relative *Clspn* expression in purified splenic B-cells from C57Bl/6 (n=3) and *Rela*^{T505A} (n=3) mice. Data represents mean ± SEM.

Figure S9:

(A) WT Eµ-Myc mice with lower Claspin levels develop lymphoma earlier. Kaplan-Meier survival analysis of WT Eµ-Myc mice comparing above and below median level expression of *Clspn* mRNA. Also with survival data from Eµ-Myc*/Rela*^{T505A} (Fig. 3A) and Eµ-Myc/*cRel-/-* mice [25].

(B) Table detailing the median survival of Eµ-Myc (high *Clspn*), Eµ-Myc (low *Clspn*) and Eµ-Myc*/Rela*^{T505A} mice

(C) Western blot analysis of CLASPIN or ACTIN in snap frozen tumour extracts prepared from re-implanted Eµ-Myc, Eµ-Myc/*cRel-/-* and Eµ-Myc*/Rela*^{T505A} tumours from inguinal lymph nodes 8 hours following a single dose of CCT244747. The data shows that the expression of CLASPIN is lost in the Eµ-Myc/*cRel-/-* tumours and reduced in Eµ-Myc*/Rela*^{T505A} tumours compared to WT Eµ-Myc. Please note the actin blot used here is replicated in another paper [40], where it is used as the control for other proteins analysed using this membrane.

(D) Western blot analysis of Claspin, ATR and CHK1 in two independent isolates of wildtype (WT) and *Clspn* +/- Ear Fibroblasts

(E) Primary fibroblasts from *Clspn*+/- mice [25] are resistant to Chk1 inhibition. Cell viability (Prestoblue assay) in wild type and Clspn+/- primary ear fibroblasts following treatment with increasing concentrations of the Chk1 inhibitor, CCT244747. This is an independent isolate of EFs to those used in Figure 8E. p* <0.05, p<0.01**, p<0.001*** (ONE-way Anova with Sidak post-hoc test).

(F) QPCR analysis showing the effect on CLSPN mRNA levels following siRNA knockdown in U2OS cells.

Supplementary data files

Supp Data File 1 Proteomics.xlsx

Data from proteomics analysis of reimplanted Eμ-Myc lymphoma cells with either vehicle of CHK1i (CCT244747) treatment for 8 hours. Please note, this data file also accompanies two other manuscripts where we use Eμ-Myc lymphoma cells [24, 40].

Supp Data File 2 RNASeq_all_genes_list_EuMyc.xlsx

Gene lists from RNA Seq analysis of reimplanted Eμ-Myc lymphoma cells with either vehicle of CHK1i (CCT244747) treatment for 8 hours. Please note, this data file also accompanies two other manuscripts where we use Eμ-Myc lymphoma cells [24, 40].

Supp Data File 3 RNASeq_counts_tximport_EuMyc.csv

Data for all genes and samples from RNA Seq analysis of reimplanted Eμ-Myc lymphoma cells. Please note, this data file also accompanies two other manuscripts where we use Eμ-Myc lymphoma cells [24, 40].

Supp Data File 4 Putative RelA target genes.xlsx

Identification of putative RelA target genes displaying altered expression in RelA T505A Eμ-Myc lymphoma cells from RNA Seq data

Supp Data File 5_Combined David analysis.xlsx

David ontogeny analysis of proteins and phosphor proteins displaying altered expression in RelA T505A Eμ-Myc lymphoma cells from proteomics data (Supp Data File 1).

Supp Data File 6_Venn Diagram data v2.xlsx

Data used to create Venn diagrams

Supp Data File 7 STRING interactions.xlsx

STRING interactions data based phosphorylated proteins identified from phospho proteomics analysis.

Supp Data File 8_RNASeq_Proteomics links to CHK1.xlsx

Analysis using String and Biogrid of genes/proteins with altered expression for previously reported direct interactions with CHK1.

Table S1

Organ	Mean cell count ± SEM (x10^6)		p-value
	WT	T505A	
Spleen	46.00 ± 3.425	44.67 ± 2.951	NS
Inguinal lymph node	2.367 ± 0.2431	3.133 ± 0.4193	

		Spleen			Blood			Inguinal lymph nodes		
Cell marker(s)	Cell population	WT Mean ± SEM	T505A Mean ± SEM	p-value	WT Mean ± SEM	T505A Mean ± SEM	p-value	WT Mean ± SEM	T505A Mean ± SEM	p-value
CD19$^+$	B cells as a % of total non-apoptotic cells	46.12 ± 2.65	41.72 ± 4.15	NS	26.46 ± 8.76	27.94 ± 3.25	NS	20.28 ± 1.42	14.91 ± 3.53	NS
CD3$^+$	T cells as a % of total non-apoptotic cells	40.75 ± 1.73	42.37 ± 3.59	NS	23.45 ± 2.90	25.08 ± 2.79	NS	75.25 ± 1.19	67.68 ± 3.13	NS
CD138$^+$/CD19$^+$	Plasma cells as a % of CD19$^+$ cells	23.73 ± 3.91	17.78 ± 2.94	NS	24.80 ± 4.00	18.82 ± 2.46	NS	33.60 ± 5.62	27.49 ± 3.06	NS
CD11b$^+$/CD19$^+$	B1 B cells as a % of CD19$^+$ cells	8.58 ± 1.43	9.12 ± 1.54	NS	34.07 ± 2.81	38.77 ± 5.30	NS	5.41 ± 0.46	6.22 ± 1.70	NS
CD11b$^+$CD19$^+$CD5$^+$	B1a B cells as a % of CD19$^+$CD11b$^+$ cells	19.90 ± 3.33	24.50 ± 6.03	NS	35.82 ± 3.95	35.52 ± 4.07	NS	-	-	-
CD11b$^+$/CD19$^+$/CD5$^-$	B1b B cells as a % of CD19$^+$ CD11b$^+$ cells	77.50 ± 4.01	73.33 ± 6.30	NS	64.23 ± 3.65	64.50 ± 3.69	NS	-	-	-
CD4$^+$/CD3$^+$	CD4 T cells as % of CD3$^+$ cells	60.17 ± 2.17	60.85 ± 0.67	NS	62.65 ± 4.33	64.98 ± 2.88	NS	52.93 ± 0.53	56.55 ± 1.95	NS
CD8$^+$/CD3$^+$	CD8 T cells as % of CD3$^+$ cells	32.88 ± 2.76	32.00 ± 1.08	NS	32.37 ± 4.59	31.30 ± 2.79	NS	45.15 ± 0.49	41.28 ± 1.83	NS
CD3$^+$/CD4$^+$/CD25$^+$	CD4$^+$CD25$^+$ cells as a % of CD3$^+$ cells	6.738 ± 0.59	6.19 ± 0.32	NS	-	-	-	4.64 ± 0.54	4.55 ± 0.55	NS
CD3$^+$/CD8$^+$/CD25$^+$	CD8$^+$CD25$^+$ cells as a % of CD3$^+$	0.45 ± 0.085	0.44 ± 0.07	NS	-	-	-	0.34 ± 0.10	0.33 ± 0.06	NS
CD4$^+$/FOXP3$^+$	T regs as % of CD4$^+$ T cells	2.44 ± 0.24	2.62 ± 0.18	NS	0.37 ± 0.08	0.46 ± 0.10	NS	4.16 ± 0.14	4.17 ± 0.24	NS
F4/80$^+$CD11c$_-$	Macrophages as a % of total no-apoptotic cells	5.65 ± 2.42	7.41 ± 2.35	NS	2.97 ± 1.67	1.66 ± 0.81	NS	0.39 ± 0.14	0.29 ± 0.07	NS
CD11c$^+$F4/80$^+$	Dentritic cells as a % of total non-apoptotic cells	2.17 ± 0.34	2.07 ± 0.28	NS	2.34 ± 0.78	2.27 ± 0.99	NS	0.95 ± 0.05	0.80 ± 0.21	NS

Table S2. Flow cytometric analysis of spleen, blood and inguinal lymph node cell populations of 6 x WT and 6 xT505A 12 week old male littermates. Peritoneal fluid control data is not shown. Data analysed using a two-tailed Students t-test.

Supp Figure 1

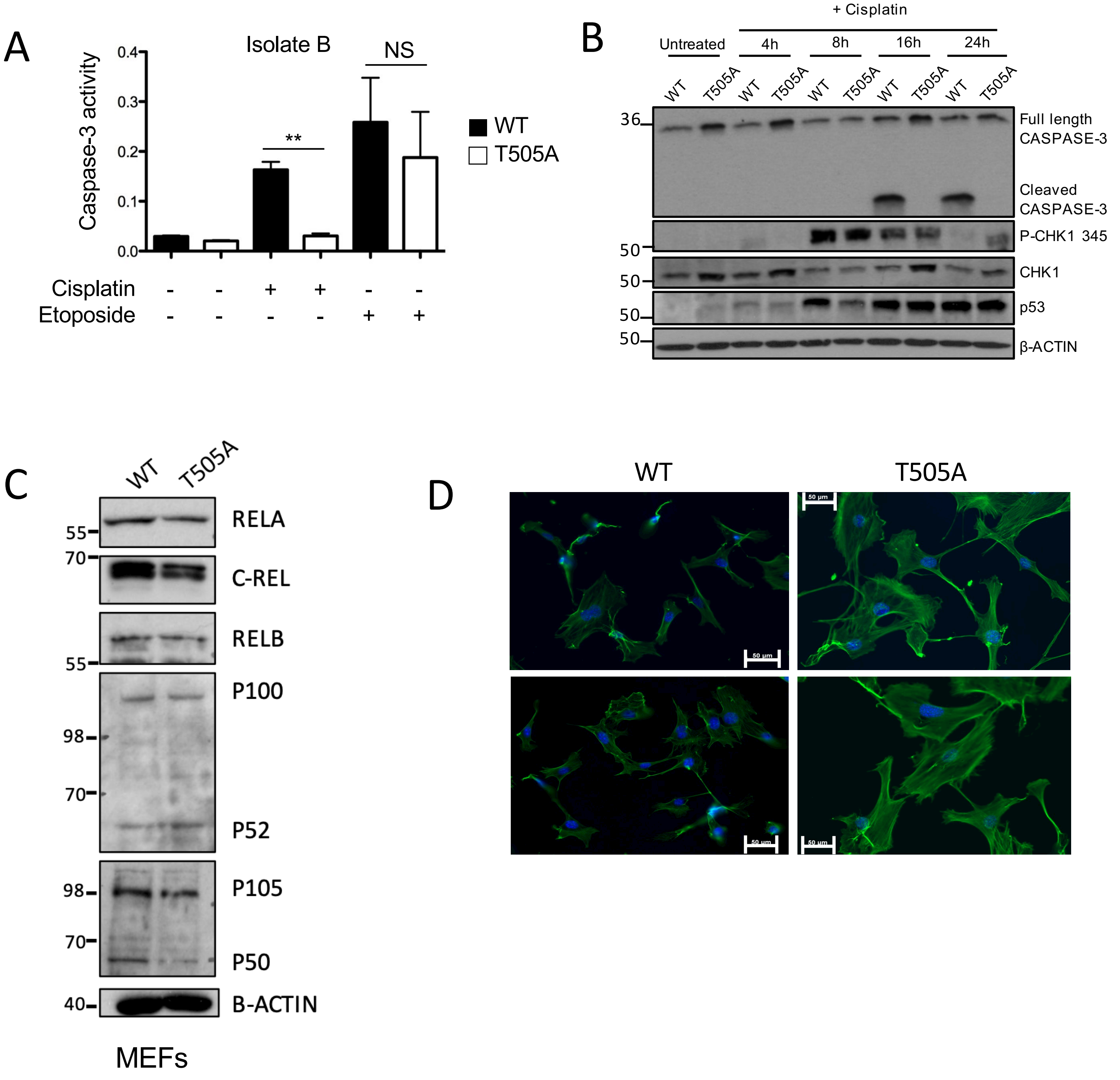

Supp Figure 2

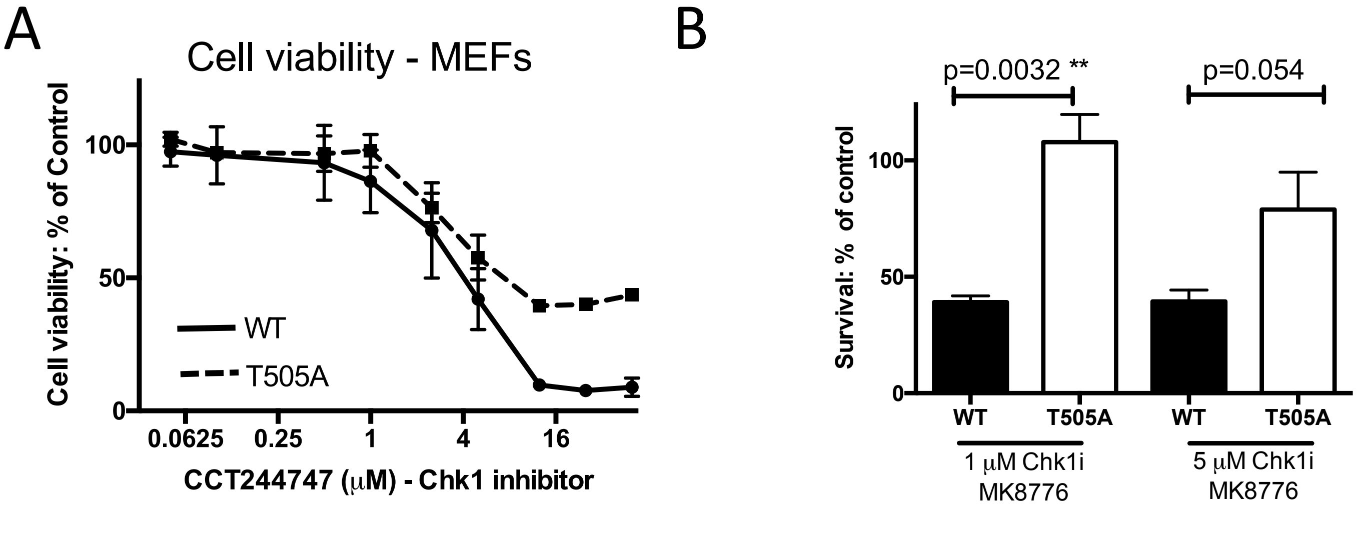

Supp Figure 3

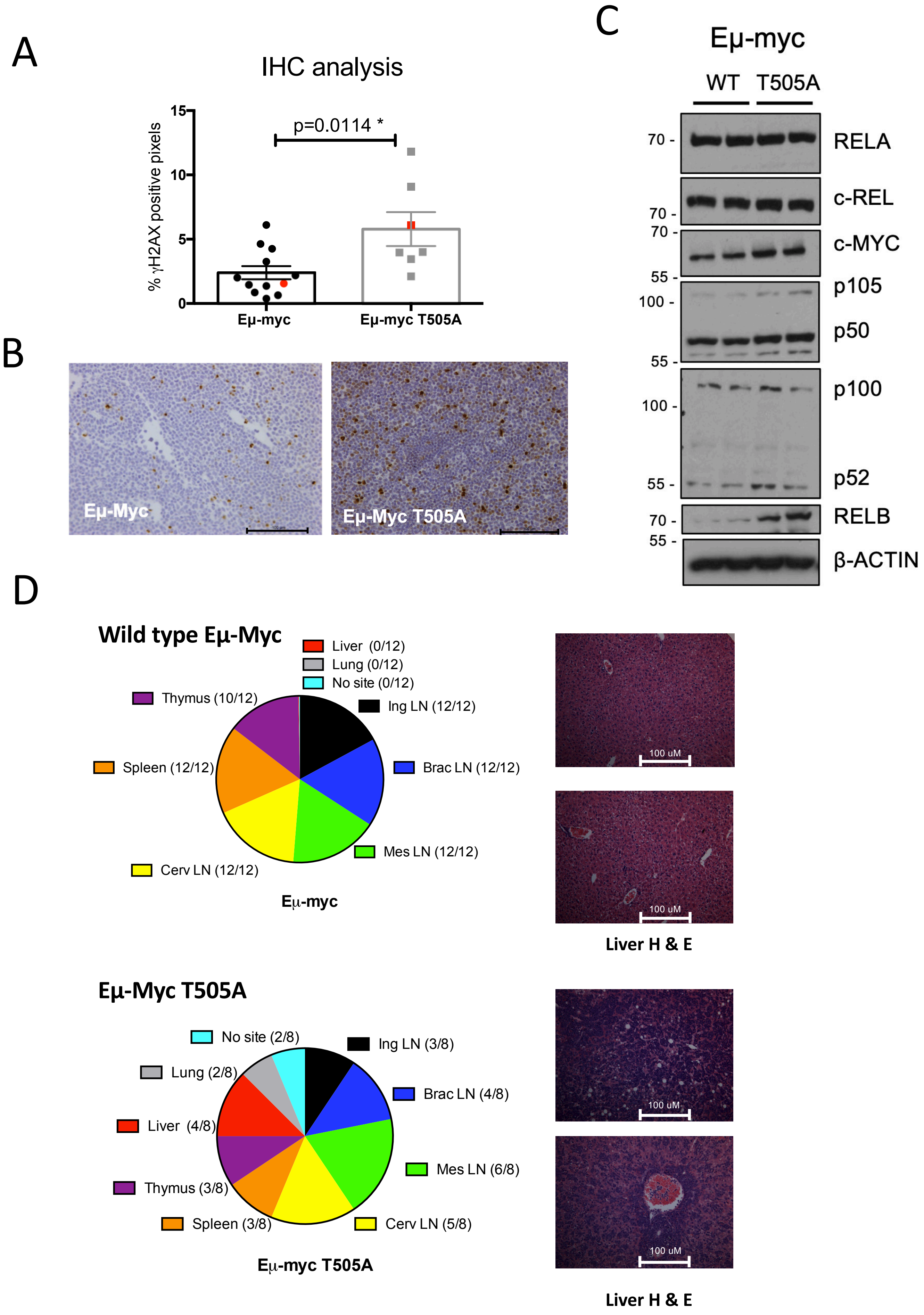

Supp Figure 4

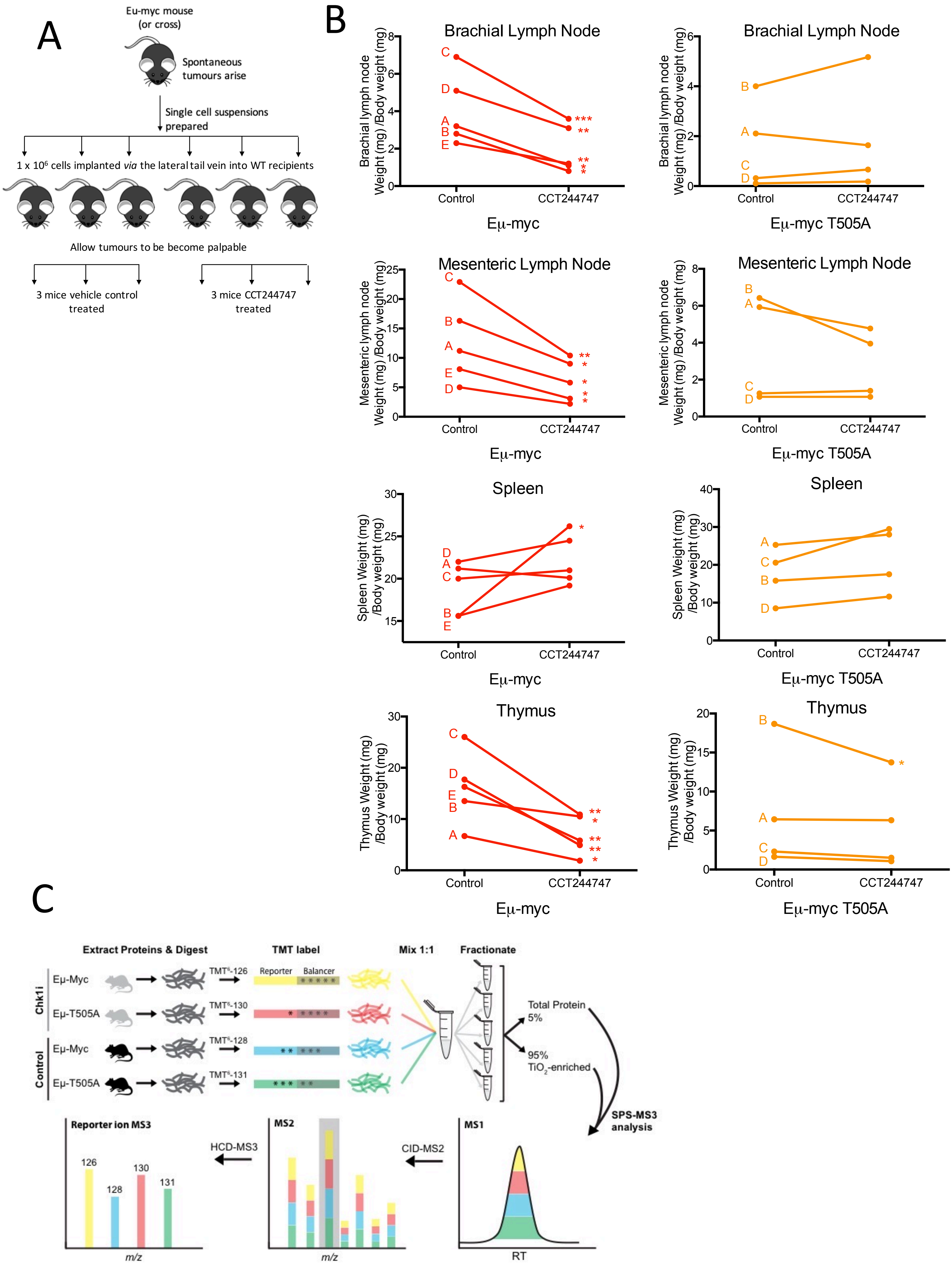

Supp Figure 5

A

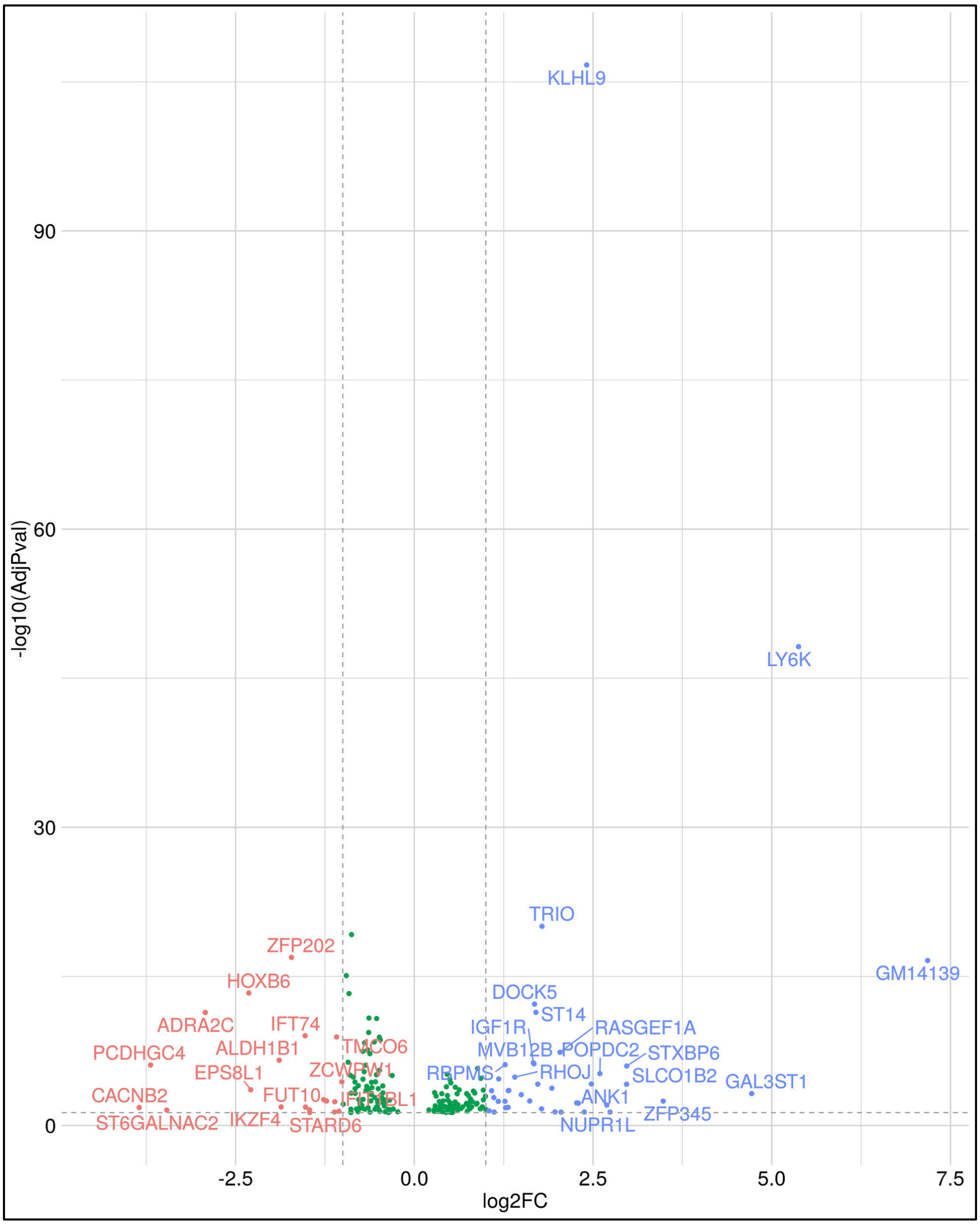

B

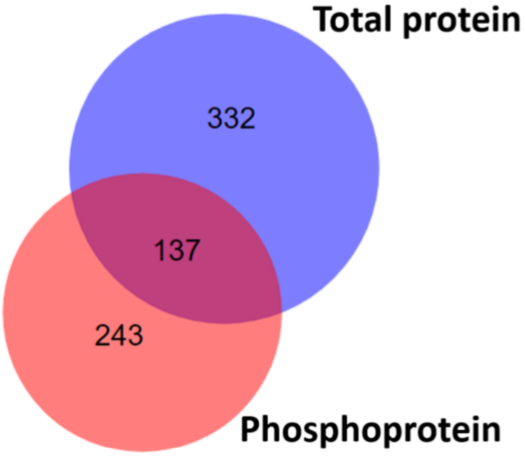

Overlap total protein vs phosphoprotein
(WT_T505, no inhibitor)

C

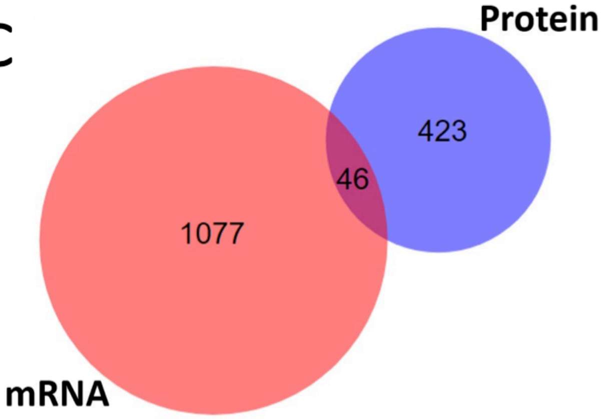

Overlap total protein vs mRNA
(WT_T505, no inhibitor)

Supp Figure 6

Phospho proteome: WT vs WT CHK1i

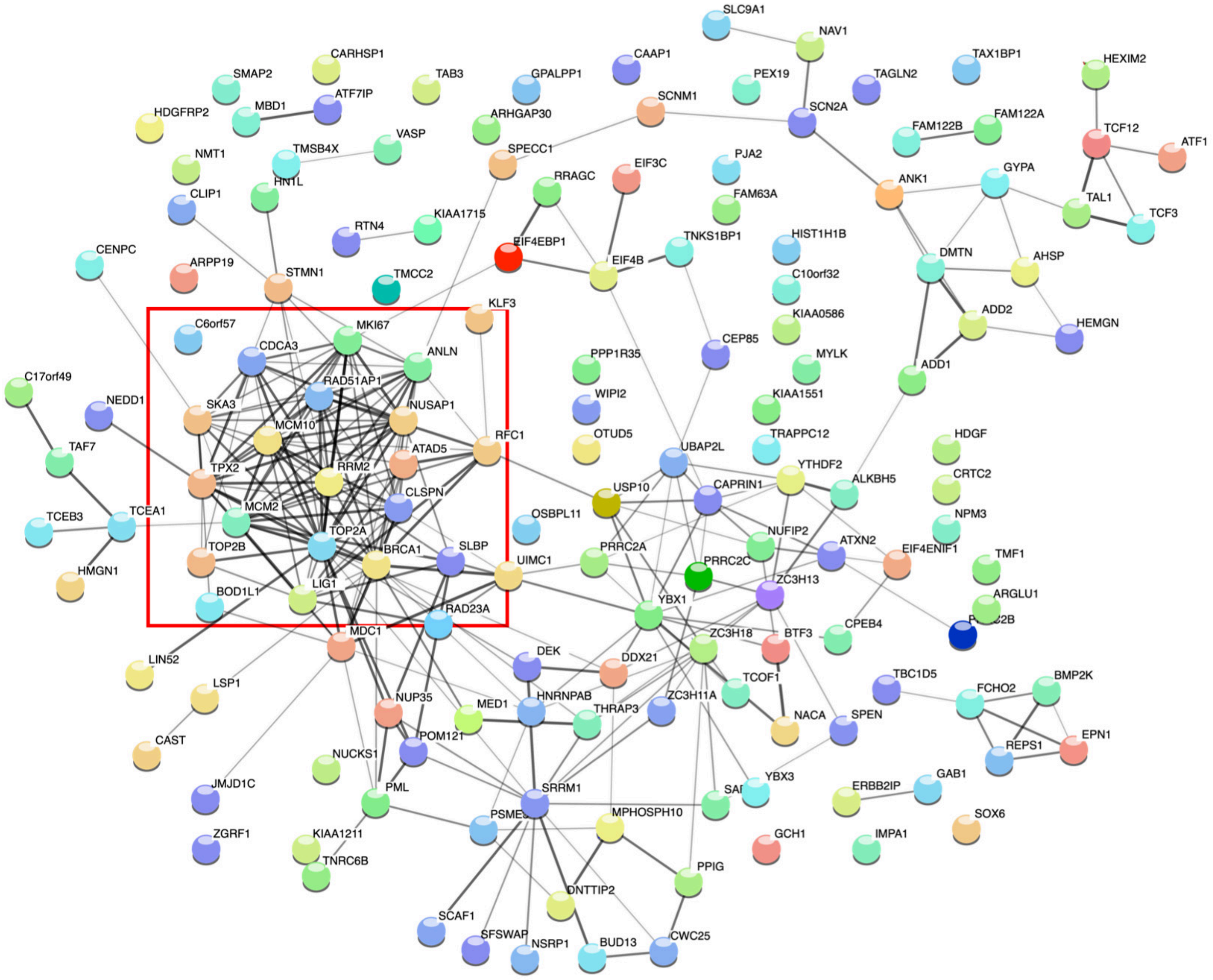

Supp Figure 7

A

symbol	logFC	adj.P.Val
Gm13414	44.5606	2.81E-27
Gm20431	48.56773	1.33E-19
Gm12350	-37.9751	4.30E-11
Gm2274	19.61534	2.08E-06
Vat1	0.494422	7.06E-05
Tsc22d1	0.836907	3.27E-04
Hist1h2bc	1.134428	0.001278
Tmem246	4.429315	0.001278
Kcnc1	3.196627	0.002925
Hist3h2a	0.670209	0.003209
Ighv1-5	4.203006	0.003728
Ighv1-85	-4.3458	0.00404
Arhgef25	1.28526	0.007336
Cd4	0.85755	0.009231
Spaca6	1.84972	0.009231
Nexn	2.549369	0.009231
Pou2f1	1.510426	0.013714
Igfbp7	0.342801	0.040663
Tspan10	0.711184	0.040663
Hist1h1c	1.108617	0.040663
Rasgef1b	0.747539	0.044182
Ighv5-12	-5.80491	0.044838
Cacna1c	1.33931	0.044838
Nupr1l	3.459597	0.044838
Plvap	0.4115	0.045762
Gm42653	1.00125	0.045762

RNA Seq WT CHK1i vs WT

B

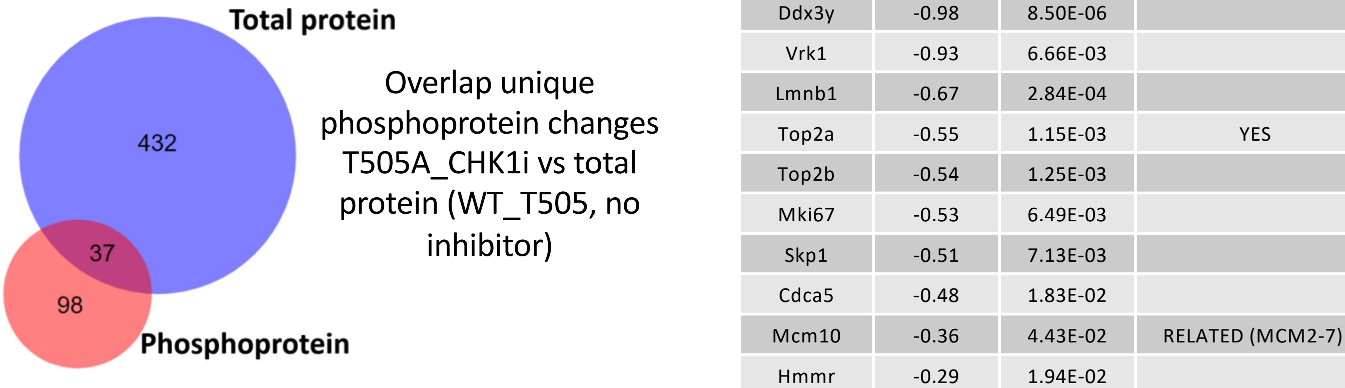

Overlap unique phosphoprotein changes T505A_CHK1i vs total protein (WT_T505, no inhibitor)

C

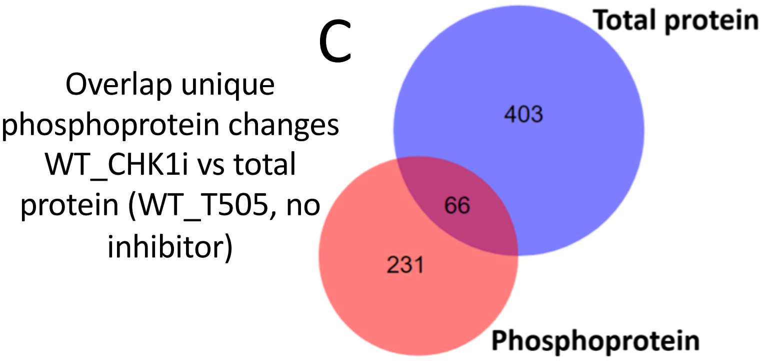

Overlap unique phosphoprotein changes WT_CHK1i vs total protein (WT_T505, no inhibitor)

D

RNASeq links to CHK1 from string (RelAT505A vs WT)			
Symbol	log2FC	pAdj	Biogrid interactor
Mgmt	-2.65	2.95E-18	
Ddx3y	-2.35	1.66E-52	
Usp1	-1.16	1.21E-08	
Poli	-0.91	5.33E-14	
Ube2d2a	-0.77	2.84E-15	
Ccnd3	-0.60	4.26E-04	
Pik3c3	-0.51	6.33E-09	RELATED (PIK3CA)
Rev3l	-0.50	1.66E-03	
Rbbp8	-0.50	2.61E-03	
Rev1	-0.50	2.52E-02	
Hjurp	-0.47	2.19E-02	
Mdm4	-0.46	2.08E-03	YES
Parp2	-0.44	6.12E-04	RELATED (PARP1)
Sirt1	-0.36	4.84E-02	
Casp8	-0.34	8.46E-08	YES
Terf1	-0.30	2.57E-02	
Nek7	-0.23	3.57E-02	RELATED (NEK2/NEK9)
E2f6	0.44	7.09E-03	
Rad51d	0.48	5.43E-08	YES
Ddb2	0.50	4.74E-03	RELATED (DDB1)
Vcl	0.62	4.46E-02	
Mcm9	0.68	3.20E-02	RELATED (MCM2-7)
Gm2423	1.79	2.85E-02	RELATED (SFN_YWHAS)
Cdk18	2.13	7.16E-04	

Total proteome links to CHK1 from string (RelAT505A vs WT)			
Symbol	log2FC	P value	Biogrid interactor
H1f0	-1.09	2.34E-03	
Ddx3y	-0.98	8.50E-06	
Vrk1	-0.93	6.66E-03	
Lmnb1	-0.67	2.84E-04	
Top2a	-0.55	1.15E-03	YES
Top2b	-0.54	1.25E-03	
Mki67	-0.53	6.49E-03	
Skp1	-0.51	7.13E-03	
Cdca5	-0.48	1.83E-02	
Mcm10	-0.36	4.43E-02	RELATED (MCM2-7)
Hmmr	-0.29	1.94E-02	
Tp53bp1	-0.26	2.38E-02	YES
Cdca2	-0.24	4.57E-02	
Ddx3x	-0.22	3.62E-02	
Sumo3	0.25	3.77E-02	
Cks2	0.27	1.61E-02	

Supp Figure 8

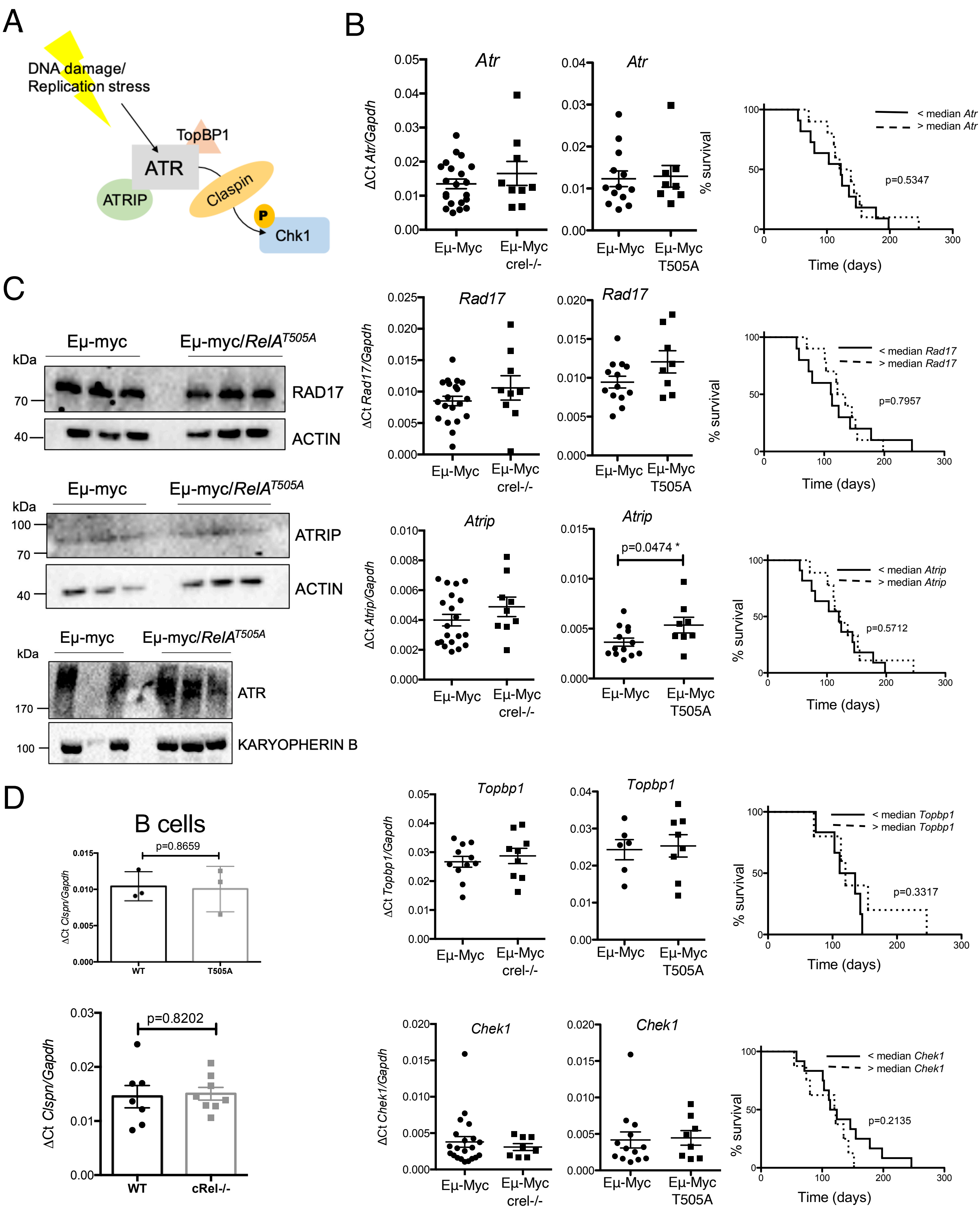

Supp Figure 9

A

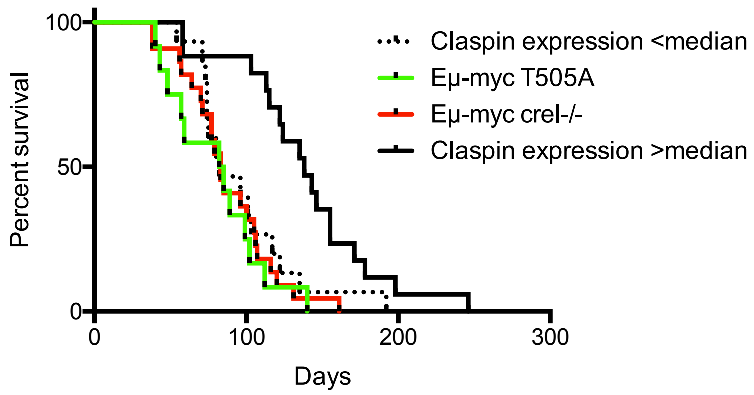

B

Cohort	Median survival
Eμ-myc >median	138 days
Eμ-myc <median	82 days
Eμ-myc T505A	83.5 days

C

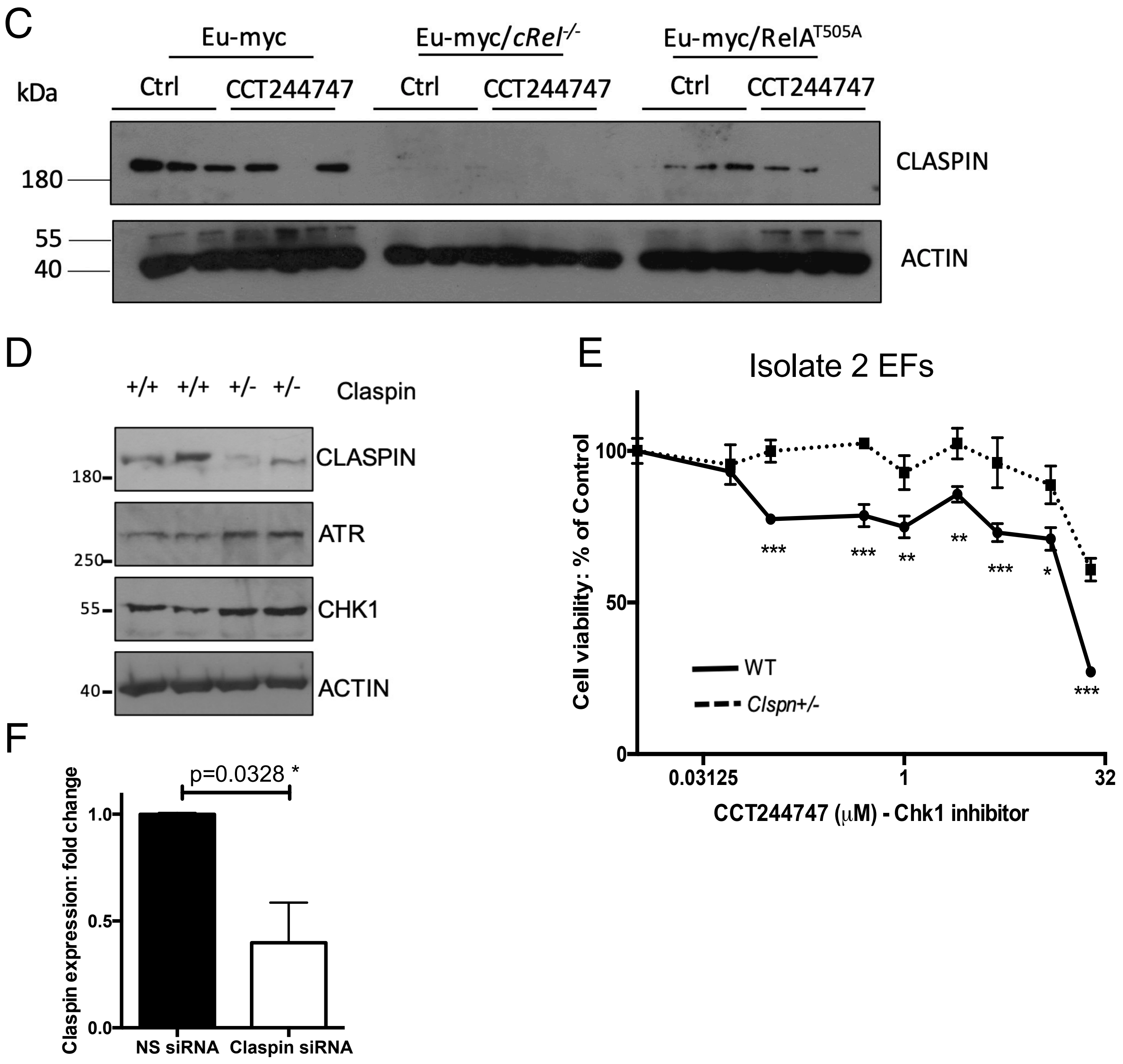

Supplement: Supplementary Material [file BCJ-479-2087-s1.pdf]
